# A pore-forming protein-induced surface-enhanced Raman spectroscopic strategy for dynamic tracing of cell membrane repair

**DOI:** 10.1016/j.isci.2021.102980

**Published:** 2021-08-14

**Authors:** Yuanjiao Yang, Yunlong Chen, Jingxing Guo, Huipu Liu, Huangxian Ju

**Affiliations:** 1 State Key Laboratory of Analytical Chemistry for Life Science, School of Chemistry and Chemical Engineering, Nanjing University, Nanjing 210023, P.R. China

**Keywords:** Membranes, Cell biology, Biophysics, Bioengineering

## Abstract

The plasma membrane repair holds significance for maintaining cell survival and homeostasis. To achieve the sensitive visualization of membrane repair process for revealing its mechanism, this work designs a perforation-induced surface-enhanced Raman spectroscopy (SERS) strategy by conjugating Raman reporter (4-mercaptobenzoic acid) loaded gold nanostars with pore-forming protein streptolysin O (SLO) to induce the SERS signal on living cells. The SERS signal obviously decreases with the initiation of membrane repair and the degradation of SLO pores due to the departure of gold-nanostar-conjugated SLO. Thus, the designed strategy can dynamically visualize the complete cell membrane repair and provide a sensitive method to demonstrate the SLO endocytosis- and exocytosis-mediated repairing mechanism. Using DOX-resistant MCF-7 cells as a model, a timely repair-blocking technology for promoting the highly efficient treatment of drug-resistant cancer cells is also proposed. This work opens an avenue for probing the plasma membrane repairing mechanisms and designing the precision therapeutic schedule.

## Highlights

• SERS strategy demonstrates the endocytosis- and exocytosis-mediated repairing mechanism
• The SLO endocytosis and exocytosis predominate at different repair stages
• SERS strategy possesses versatile applicability for monitoring the repairing process
• Prompt blocking of the repair process can promote effective treatment of cancer cells

## Graphical abstract

**Figure:**
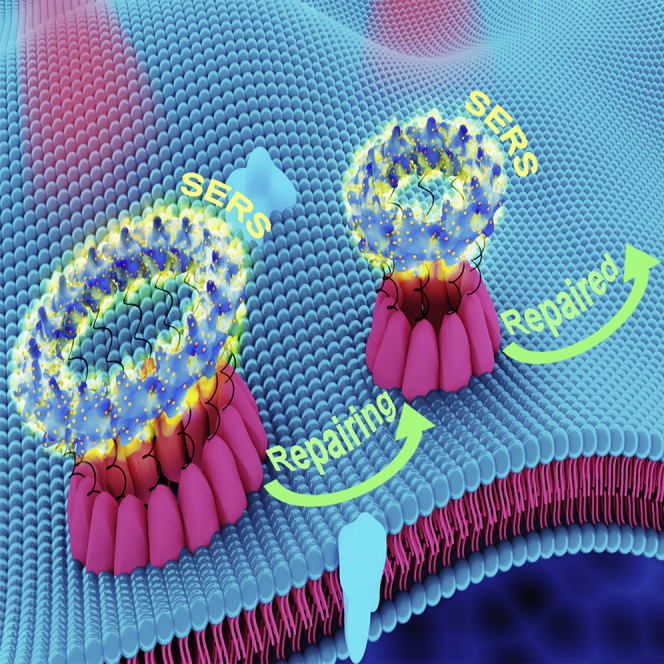

## Introduction

Membrane pores are ubiquitous on cell surface, which are associated with cell death in immune system, inflammation, or related diseases (Baran et al., 2009; Ding et al., 2016; Mellgren et al., 2007). Typically, perforins secreted by natural killer cells and T cells can form membrane pores for delivery of granzymes into the immune synapse to induce cell death (Law et al., 2010). These membrane pores are also produced when cells are exposed to some exogenous threats. Streptolysin O (SLO), an oxygen-labile bacterial exotoxin belonging to cholesterol-dependent cytolysin family (Alouf, 2000; Gilbert, 2002; Morton et al., 2019), can bind to cholesterol of the cell membrane and oligomerize into large pores (Palmer et al., 1998b; Abdel Ghani et al., 1999) and thus has been used to probe the mechanism of pore formation (Liu et al., 2018; Palmer et al., 1998a; Tilley et al., 2005; Feil et al., 2014). Despite SLO has different structures and shows various pore-forming mechanisms, it can be used to replace perforin to form pores and facilitate granzyme-induced cell death (Lieberman, 2003; Browne et al., 1999). It has been well known that the timely initiation of pore-repairing process can block membrane injury and resist the entry of extracellular species for maintaining cellular homeostasis and ensuring cell survival (Jimenez and Perez, 2017; Brito et al., 2019). However, while the pore-forming mechanism is well studied, the accurate monitoring and mechanism study of pore repair on plasma membrane is difficult to achieve due to the lack of sensitive technology. In order to explore the repairing mechanisms and ultimately design the precision therapeutic schedule, it is very necessary to develop the visualization method for dynamic tracing of cell membrane repair.

The visualization of membrane pores is mainly performed on the fixed cells or artificial lipid bilayers through electron microscopy and atomic force microscopy (AFM). For electron microscopy, the destructive pretreatment and vacuum system prevents its application on living cell (Leung et al., 2014; McNeil and Baker, 2001). AFM provides a noninvasive imaging of living cell with nanometer resolution, but the mobility of living cells limits the effective contact of the AFM tip with membrane. Thus, AFM is inapplicable to continuously trace the dynamic processes occurring on membrane, such as the dynamic repair process of injured membrane (Liu et al., 2018; Leung et al., 2017). Some fluorescence methods have also been used to study the processes and mechanisms of membrane repair by using Ca^2+^-sensitive fluorescent dyes to label the Ca^2+^ influx across membrane pores and verifying the repair with the Ca^2+^-dependent behavior (Idone et al., 2008; Babiychuk et al., 2009). However, the indirect determination of the repair process through Ca^2+^ influx may miss the synchronization between fluorescence intensity (FI) readout and the actual repairing process. In addition, the methods based on immunofluorescence or fluorescent encoding of pore-forming monomers (Romero et al., 2017) are insufficient to dynamically monitor the repair process or accurately reveal the repairing mechanism due to the limited sensitivity.

**Figure 1:**
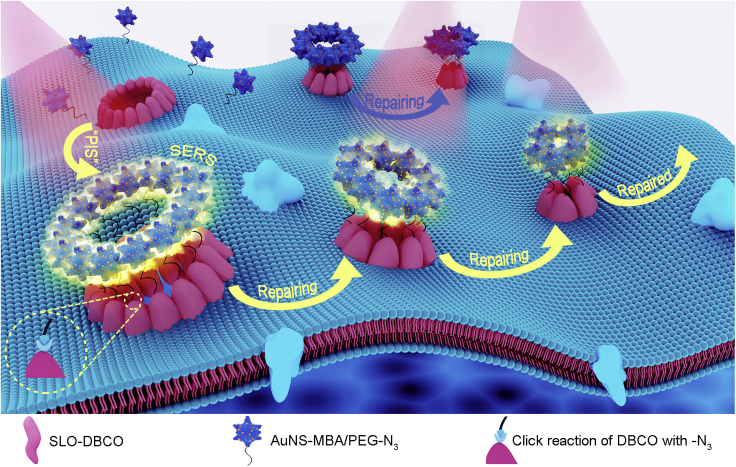
Schematic illustration of the pore-forming protein-induced SERS (PIS) strategy for dynamic monitoring of cell membrane repair After the perforation and aggregation of DBCO modified pore-forming protein (SLO) on living cell membrane, the azido-polyethyleneglycol (PEG-N_3_) stabilized SERS gold nanostars (AuNSs) are conjugated to generate the SERS signal. The initiation of membrane repair along with the degradation of SLO-pores leads to the departure of conjugated AuNSs and thus decreases the SERS signal for dynamically revealing the entire repairing process of membrane.

As a powerful technology competitive with fluorescence imaging, surface-enhanced Raman spectroscopic (SERS) imaging can provide biochemical information and study complex biological processes due to its high sensitivity (Abramczyk and Brozek-Pluska, 2013; Zong et al., 2018). Thus, this work designed a pore-forming protein-induced SERS (PIS) strategy for noninvasive and highly sensitive Raman imaging of pore-repairing process on living cell membrane (Figure 1). As a proof-of-concept, SLO was chosen as the model of pore-forming proteins, which was coupled with DBCO-sulfo-NHS ester to obtain SLO-DBCO. After *in situ* formation of membrane pores by SLO-DBCO, the HS-PEG-N_3_ (PEG-N_3_) functionalized gold nanostars (AuNSs) loaded with 4-mercaptobenzoic acid (MBA) as a Raman reporter were subsequently bound to the pores through copper-free click chemistry (Sletten and Bertozzi, 2009). Considering the efficient enhancement of Raman signal by AuNSs and their steric hindrance to complete binding of SLO-DBCO on living cells, 24-nm AuNSs were selected for the labeling. By dynamic Raman imaging of the living cells, the pore-repairing process could be visualized. The proposed strategy showed the endocytosis and exocytosis signals of the pore-forming proteins during the repairing process, thus providing a sensitive protocol for the first time to trace the intact repair process and mechanism of the plasma membrane injured by pore-forming protein.

## Results

### Perforating ability of SLO-DBCO

**Figure 2:**
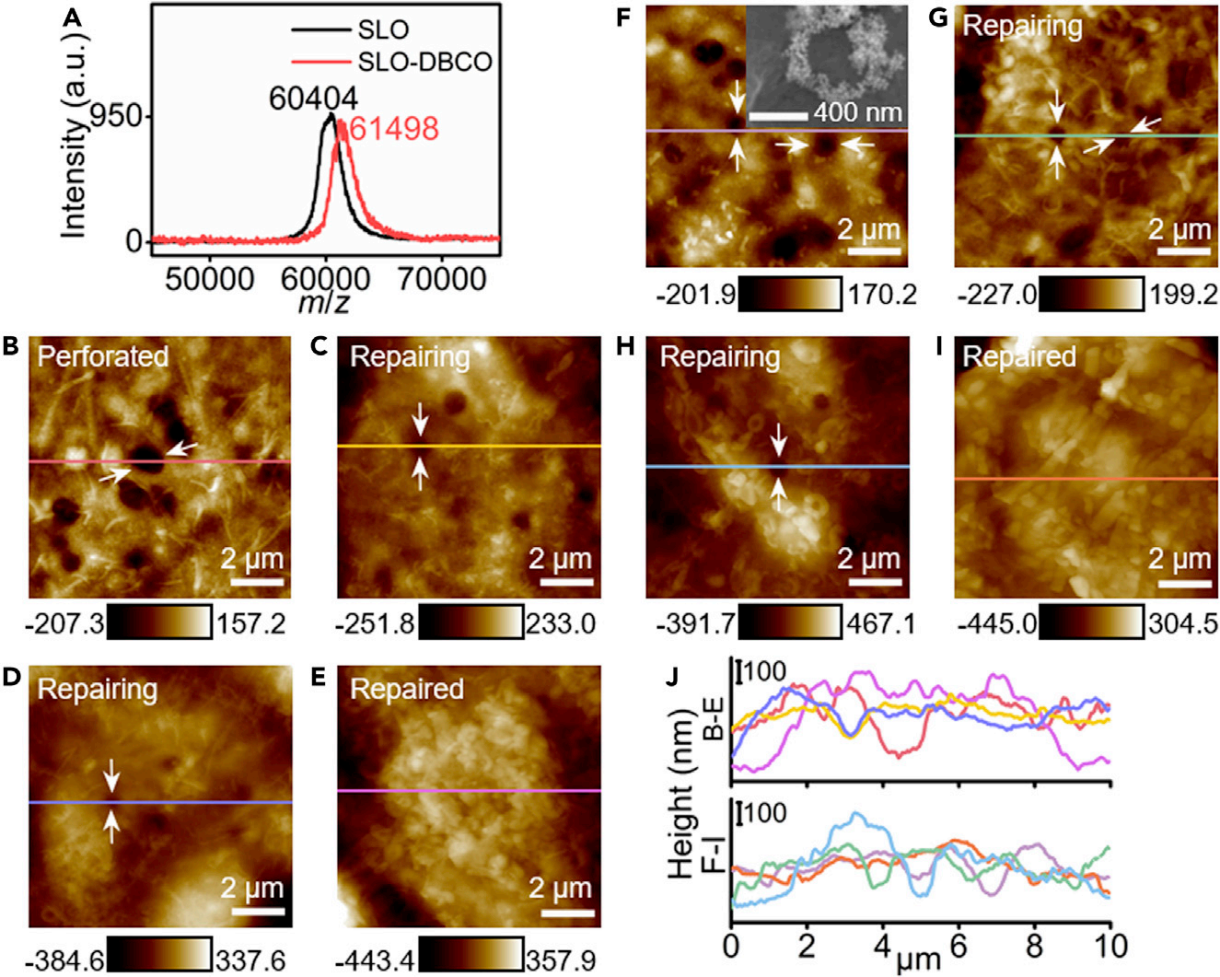
Verification of the perforating ability of SLO-DBCO and repairable ability of SLO-DBCO perforated cell membrane before or after decoration of AuNSs (A–E) (A) MALDI-TOF mass spectra of SLO and SLO-DBCO. AFM topography of SLO-DBCO perforated MCF-7 cell membrane (B) before and after repair for 40 (C), 60 (D) and 90 (E) min in 10% FBS-containing RPMI-1640. White arrows indicate some typical pores. (F–I) (F) AFM image of MCF-7 cell membrane perforated with SLO-DBCO and then decorated with AuNSs-MBA/PEG-N_3_. Inset: SEM image of a single pore therein. AFM image of treated cell F after repaired in 10% FBS-containing RPMI-1640 for 40 (G), 60 (H) and 90 (I) min. (J) Height profiles corresponding to the lines with the same colors in (B–E) and (F–I).

SLO-DBCO was firstly characterized with mass spectroscopy, which exhibited an obvious increase of m/z from 60,404 to 61,498 compared to SLO (Figure 2A), indicating that each SLO bound two DBCO molecules. The perforating ability of SLO was verified by 2D and 3D AFM imaging. After the MCF-7 cells were treated with SLO (100 U mL^−1^) for 10 min, obvious pores were observed (Figures S1A–S1D). These pores showed an average diameter of 549 ± 41 nm and an average depth of 96 ± 16 nm, which could be obviously distinguished from the depression on cell membrane (~10 nm) (Liu et al., 2018). The pores could be repaired through incubating the perforated cells in 10% FBS-containing RPMI-1640 for 90 min (Figures S1E and S1F) (Giles et al., 1998). The perforating ability of SLO-DBCO was verified by the protein hemolysis assays using human erythrocytes (Figure S2). At the same dose and incubation time, the supernatant collected from the SLO-DBCO incubation exhibited weaker absorption peak of hemoglobin at 541 nm than that from the SLO incubation. After the dose was increased from 100 U mL^−1^ of SLO to 200 U mL^−1^ of SLO-DBCO and the incubation time was twice extended, the two supernatants showed similar absorption peak intensity at 541 nm, indicating the similar perforating effect. Meanwhile, the membrane pore formed with SLO-DBCO was further demonstrated by AFM imaging, which showed an average depth of 103 ± 8 nm (Figures 2B and 2J). By incubating the cells in 10% FBS-containing RPMI-1640, the pores formed by SLO-DBCO could be repaired gradually with less number and smaller size of pores and finally complete disappearance at 90 min (Figures 2C–2E and 2J).

The dose and incubation time of SLO-DBCO for cell perforation were optimized to be 200 U mL^−1^ and 20 min for MCF-7 cells, respectively, which were performed by flow cytometric assay and confocal laser scanning microscopic (CLSM) imaging with propidium iodide staining (Figures S3 and S4).

### Characterization of AuNSs-MBA/PEG-N_3_ and optimization of Raman excitation wavelength

**Figure 3:**
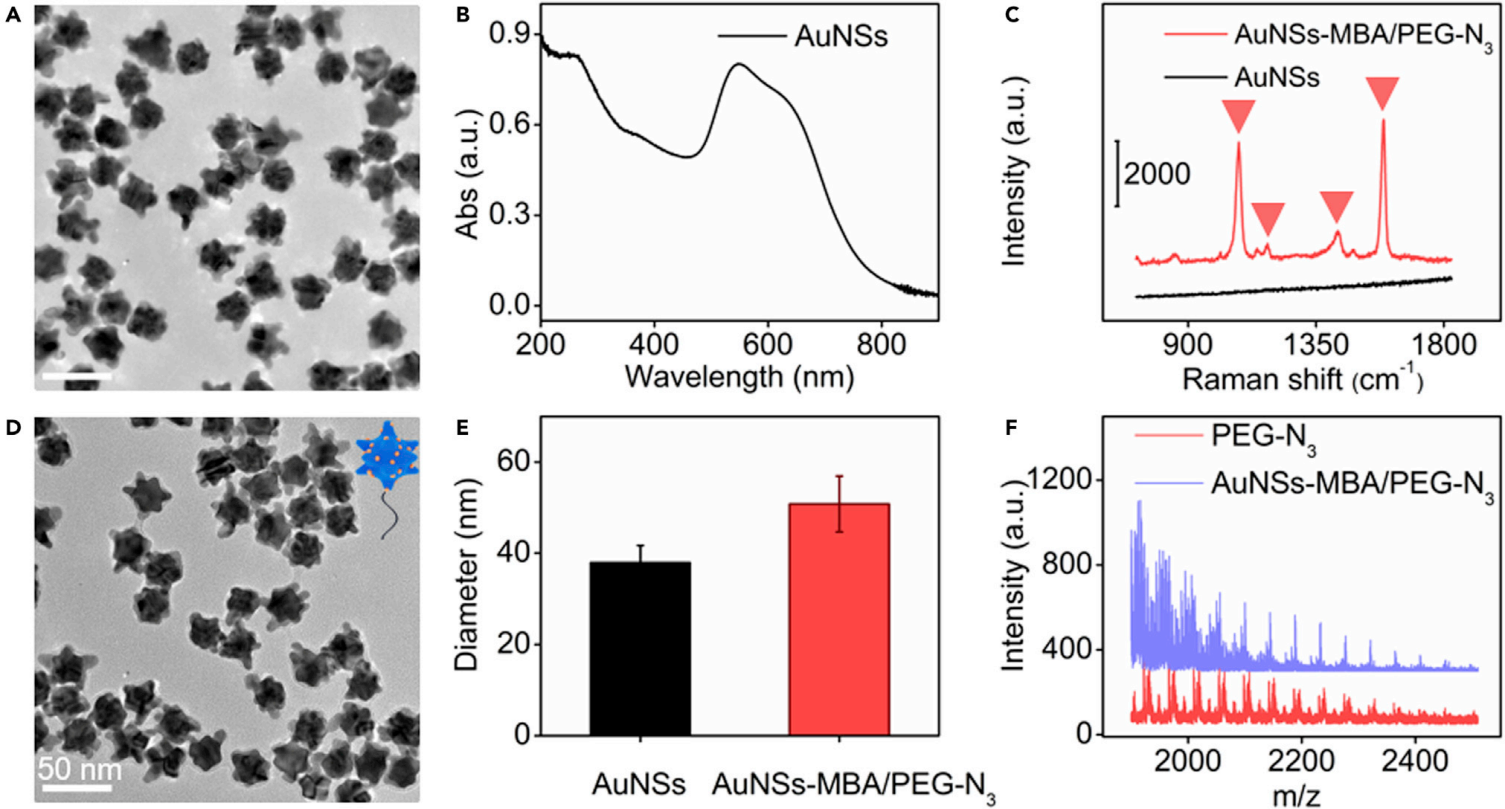
Characterization of AuNSs-MBA/PEG-N_3_ (A and B) (A) TEM image and (B) UV-vis absorption spectrum of AuNSs. (C) Raman spectra of AuNSs and AuNSs-MBA/PEG-N_3_. (D) TEM image of AuNSs-MBA/PEG-N_3_. (E) DLS analysis of AuNSs and AuNSs-MBA/PEG-N_3_. (F) MALDI-TOF mass spectra of PEG-N_3_ and AuNSs-MBA/PEG-N_3_.

The transmission electron microscopic (TEM) image and UV-Vis spectrum demonstrated the successful synthesis of AuNSs with a diameter of 24.4 ± 2.1 nm (Figures 3A and 3B) (Kumar et al., 2008). After AuNSs were modified with MBA and PEG-N_3_, the obtained AuNS-MBA/PEG-N_3_ exhibited obvious Raman characteristic peaks of MBA (Khoury and Vo-Dinh, 2008) and low background (Figure 3C), which indicated the successful loading of MBA. The modified AuNSs retained their original morphology with slightly increased size (Figure 3D), which was verified by dynamic light scattering analysis (Figure 3E). Both AuNS-MBA/PEG-N_3_ and PEG-N_3_ showed the same equally spaced *m/z* values of 44, indicating the successful modification of PEG-N_3_ on AuNSs (Figure 3F).

The cellular toxicity of AuNSs-MBA/PEG-N_3_ was evaluated with CCK8 assay. The SLO-DBCO perforated cells exhibited tiny decrease of cell viability comparing to untreated cells, while the cell viability obviously decreased after they were incubated with AuNSs-MBA/PEG-N_3_ at the concentrations higher than 0.3 nM for SLO-DBCO perforated cells and 0.5 nM for untreated cells (Figure S5). Meanwhile, AuNSs-MBA/PEG-N_3_ also showed the endocytosis in SLO-DBCO perforated cells when their concentration was higher than 0.3 nM (Figure S6). Thus, 0.2 nM of AuNSs-MBA/PEG-N_3_ was used for following experiments.

The maximum absorbance of AuNSs occurred in the range of 510–650 nm (Figure 3B), which demonstrated strong Raman signal of AuNSs-MBA/PEG-N_3_ under excitation of 633 nm (Figures S7A and S7B). However, both MCF-7 cells and SLO-DBCO perforated MCF-7 cells after incubation with AuNSs-MBA/PEG-N_3_ exhibited higher cell viability after exposure to 785 nm than 633 nm for different Raman mapping times (Figure S8). In addition, live cell staining assay also showed that 785-nm laser led to tiny cell damage, which made the fluorescence of calcein-AM-stained MCF-7 cells stronger than that exposed to 633-nm laser (Figure S9). Considering only 20% decrease of Raman signal under excitation of 785 nm, compared to that under excitation of 633 nm, and the sensitivity sufficient for monitoring the pore-repairing process, 785-nm excitation was chosen in the subsequent experiments. The aggregation of AuNSs-MBA/PEG-N_3_ was negligible after they were dispersed in the incubation media. Moreover, after MCF-7 cells and SLO perforated MCF-7 cells were incubated with AuNSs-MBA/PEG-N3, their Raman images did not show any signal (Figure S10), which excluded their nonspecific adsorption or aggregation on cell surface.

### Membrane repair after conjugation of AuNSs to membrane pores

The copper-free click reaction between PEG-N_3_ and DBCO was validated from the occurrence of the mass spectroscopic signals at m/z higher than 2500 (Figure S11), which came from the binding of DBCO to each unit of PEG. To further validate the click reaction between AuNS-MBA/PEG-N_3_ and SLO-DBCO on cells surface, MBA and HS-mPEG modified AuNSs (AuNSs-MBA/PEG) were prepared as negative control. Comparing to the strong Raman signal observed on the cells treated with SLO-DBCO and then AuNS-MBA/PEG-N_3_, the cells treated with SLO and then AuNS-MBA/PEG-N_3_ (Control-1) or SLO-DBCO and then AuNS-MBA/PEG (Control-2) exhibited negligible Raman signal (Figure S12). The phenomenon corroborated the click-reaction-mediated specific recognition of AuNS-MBA/PEG-N_3_ to SLO-DBCO treated cells with negligible nonspecific adsorption or endocytosis.

After AuNSs-MBA/PEG-N_3_ were bound to the SLO-DBCO perforated cells, the cell morphology did not obviously change because their size was much smaller than the roughness of cell membrane (Figures 2F and 2J).The SEM image exhibited the presence of AuNSs surrounding the pores (inset in Figure 2F). Moreover, the pores bound with AuNSs-MBA/PEG-N_3_ also became smaller and less and disappeared after incubating the cells in 10% FBS-containing RPMI-1640 for 90 min (Figures 2G–2I and 2J), which indicated that the conjugate of AuNSs to membrane pores had negligible effect to cell membrane repair due to the separation of SERS nanotag and SLO by the PEG2000 linker, thus maximally avoiding the influence of protein reactivity in the repairing process.

### Raman imaging of dynamic membrane repair process

As the perforated cells cannot be repaired in FBS-free medium (Giles et al., 1998), the SLO-DBCO- and then AuNSs-MBA/PEG-N_3_-treated cells were cultured in FBS-free medium for 90 min to examine the stability of AuNSs-MBA/PEG-N_3_ bound on the cells by dynamic Raman imaging, which exhibited tiny changes of the Raman signals on cell surface, and no signal could be seen in cells (Figure S13). Thus, the AuNSs-MBA/PEG-N_3_ were not released from the cell membrane into the medium and internalized to cytoplasm, which ensured the stability of AuNS-conjugated SLOs for tracing the cell repairing process.

**Figure 4:**
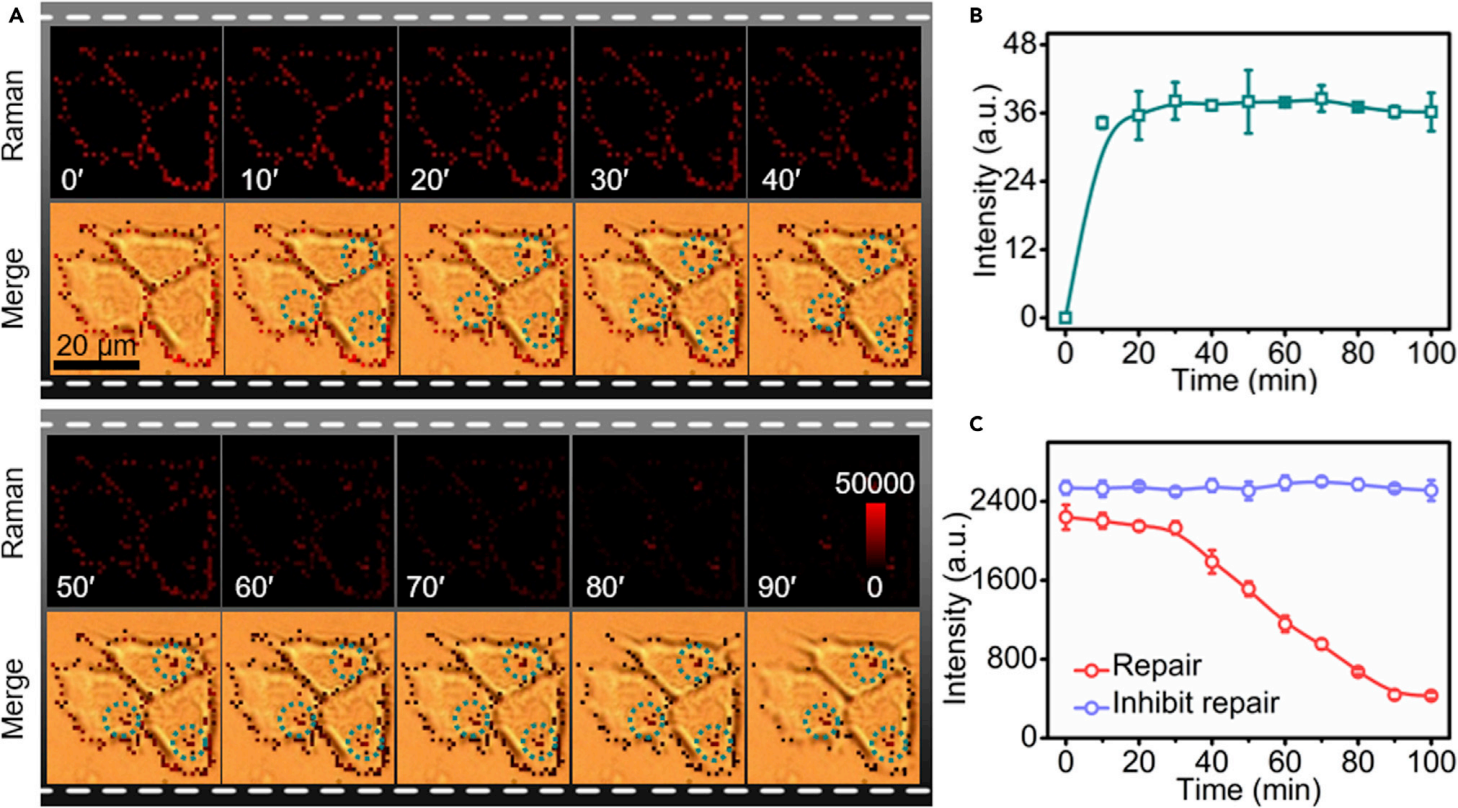
Raman imaging monitor the dynamic repair process of perforated cells (A) Dynamic Raman imaging of SLO-DBCO perforated MCF-7 cells after bound with AuNSs-MBA/PEG-N_3_ upon incubation in FBS-containing RPMI-1640 for 0–90min and washing every 10 min. The green circles show the Raman signals in cells. (B) Statistic signal intensity in cells during the repairing process. (C) Dynamic Raman intensity on cell surface for the repairing process (red line) and inhibition test to the repairing (purple line).

The repairing process of perforated cell membrane in 10% FBS-containing RPMI-1640 was traced in every 10 min by dynamic confocal Raman imaging along with the merge image with cell bright-field photo of the focal plane. The Raman signal emitted from cell surface decreased very slowly in the first 30 min. Afterward, the signal continuously reduced and reached the minimum value at 90 min (Figures 4A and 4C), indicating the leave of AuNS- bound SLO from cell membrane induced the repair of cell membrane, and the complete repair of the perforated cell membrane was achieved at 90 min. Interestingly, the repaired cells could be perforated by SLO-DBCO again (Figure S14A). Furthermore, the same Raman signal could be observed upon binding of AuNSs-MBA/PEG-N_3_ (Figure S14B), indicating the vigorous viability of the repaired cells.

Considering that the repair of SLO-induced pores was critically Ca^2+^ dependent (Idone et al., 2008; Babiychuk et al., 2009), the inhibition test was performed by incubating the AuNSs-MBA/PEG-N_3_-bound cells in Ca^2+^-free RPMI-1640 containing 10% FBS, which showed the constant Raman signal within 90 min (Figure 4C), demonstrating the correlation of the repair to the decreased Raman signal.

### Intact repairing mechanisms of injured membrane

**Figure 5:**
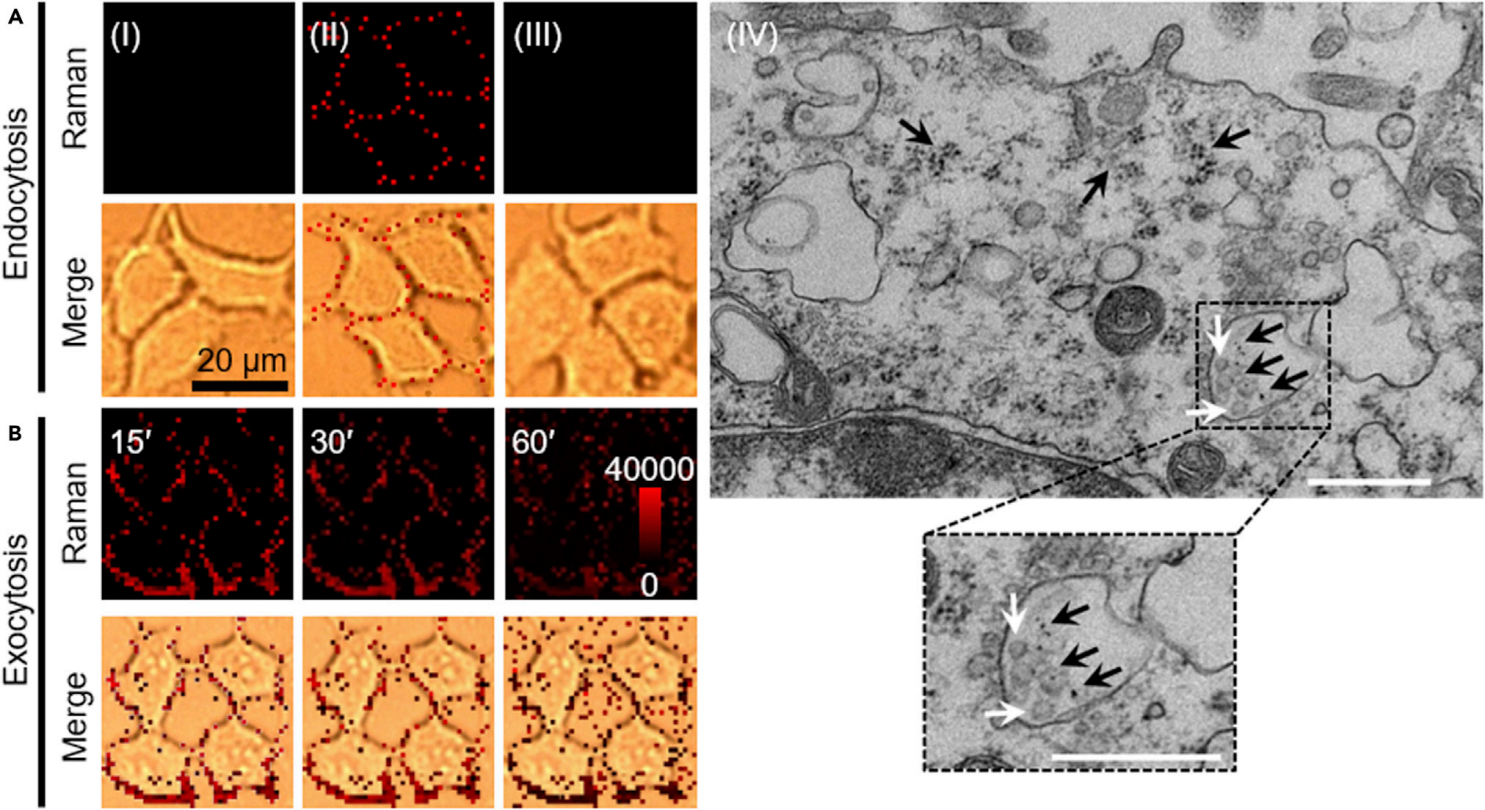
Verification of the endocytosis and the exocytosis during repair process (A) Raman imaging of (I) MCF-7 cells after incubation with AuNSs-MBA/PEG-N_3_ for 30 min, and (II, III) SLO-DBCO perforated MCF-7 cells after (II) binding AuNSs-MBA/PEG-N_3_ and then incubation in FBS-free RPMI-1640 for 90 min, and (III) repairing for 10 min and then incubation with 0.2 nM AuNSs-MBA/PEG for 10 min (IV) TEM images of the repaired MCF-7 cells after perforation by SLO-DBCO and reaction with AuNSs-MBA/PEG-N_3_. Black arrows show AuNSs trapped inside the vesicles or released to cell. White arrows show endosomes. Scale bars, 500 μm. (B) Raman imaging of SLO-DBCO perforated MCF-7 cells after binding AuNSs-MBA/PEG-N_3_ and then repairing for 15, 30 and 60 min without washing.

Different from the unrepairable process in FBS-free RPMI-1640 (Figure S13), the Raman signal emerged inside the cells after repairing for 10 min, which gradually increased in the first 30 min and then remained at a stable value until the end of repair (Figure 4B). Although the Raman signal emerging inside cells was relatively weak, this result implied the repairing process involved the endocytosis of AuNSs-MBA in the initial stage within 30 min (Idone et al., 2008; Babiychuk et al., 2009; Corrotte et al., 2012, 2013; Castro-Gomes et al., 2016). The endocytosis mechanism of the repairing process was further verified by Raman imaging (Figures 5A and 5AI–5AIII). After incubating the unperforated cells with AuNSs-MBA/PEG-N_3_ for 30 min, no Raman signal was observed on cells (Figures 5A and 5AI), indicating that the cells did not uptake the AuNSs. However, the SLO-DBCO-perforated MCF-7 cells showed obvious Raman signal after binding AuNSs-MBA/PEG-N_3_ and then incubated in FBS-free RPMI-1640 for 90 min (Figures 5A and 5AII). Moreover, the signal only occurred on cell surface, illustrating that the perforated cells did not also internalize the AuNSs during binding and incubation process. These results indicated that the involved endocytosis of the AuNSs in 10% FBS-containing RPMI-1640 resulted from the repair of perforated cell membrane instead of AuNSs passing through the large pores into cells. The endocytosis of AuNSs was further demonstrated by the TEM images of the repaired cells (Figures 5A and 5AIV), which showed that AuNSs were indeed trapped in vesicles of endosomes after the repair of perforated cell.

To trace the whereabouts of other AuNSs, the AuNSs-MBA/PEG-N_3_-bound cells were incubated in 10% FBS-containing RPMI-1640 to observe the Raman signals at 15, 30, and 60 min without washing. As shown in Figure 5B, the gradually increasing Raman signal occurred outside the cells while Raman signal on cell surface attenuated, indicating the exocytosis of the conjugates of AuNSs-MBA/PEG-N_3_ with SLO-DBCO during the repairing process (Babiychuk et al., 2011; Keyel et al., 2011). Moreover, the exocytosis of the conjugates was a major role in repairing process after 30 min. More importantly, the exocytosed conjugates could not be internalized into the cells in repairing medium, which was demonstrated by the fact that no Raman signal was observed after incubating the incompletely repaired cells with AuNSs-MBA/PEG (Figures 5A and 5AIII).

### CLSM imaging of dynamic membrane repair process

The repairing process of the perforated cells was verified by dynamic fluorescence imaging, where AF647 was conjugated to SLO for directly obtaining the fluorescence signal. When the cells perforated with SLO-AF647 were incubated in FBS-free medium, the FI on cell surface exhibited tiny change (Figure S15), indicating the cells were not repaired. After the perforated cells were incubated in 10% FBS-containing medium and imaged every 10 min with or without culture medium exchange, the cell surface showed similarly continuous decrease of FI (Figures S16 and S17), which indicated that the behavior of culture medium exchange did not affect the process of membrane repairing. The FI presented weak variation at the first 30 min. However, the intracellular fluorescence signal of AF647 was not found, which could be attributed to the related low sensitivity of fluorescence imaging. Thus, the designed Raman imaging method provided a powerful technology with obvious advantage over fluorescence imaging for revealing the repairing mechanism, and the time sequential endocytosis and exocytosis of the AuNS-bound SLO were the main roles in the membrane repair process.

### Universality and therapeutic application of Raman imaging strategy for monitoring repair process

The PIS strategy was further applied to dynamic tracing of cell membrane repair for other cancer cells such as HepG2 and HeLa. Similarly, the repairing process of perforated HepG2 and HeLa cells showed a small amount of endocytosis of the AuNSs-MBA at the first 20 min, which could be stable in the cytoplasm, and continuous decrease of Raman signal on cell surface (Figure S18). The repair of perforated HepG2 cells could almost be completed after incubating them in 10% FBS-containing RPMI-1640 for 60 min. Thus, the designed strategy possessed the versatile applicability for monitoring the repairing process of cancer cells.

**Figure 6:**
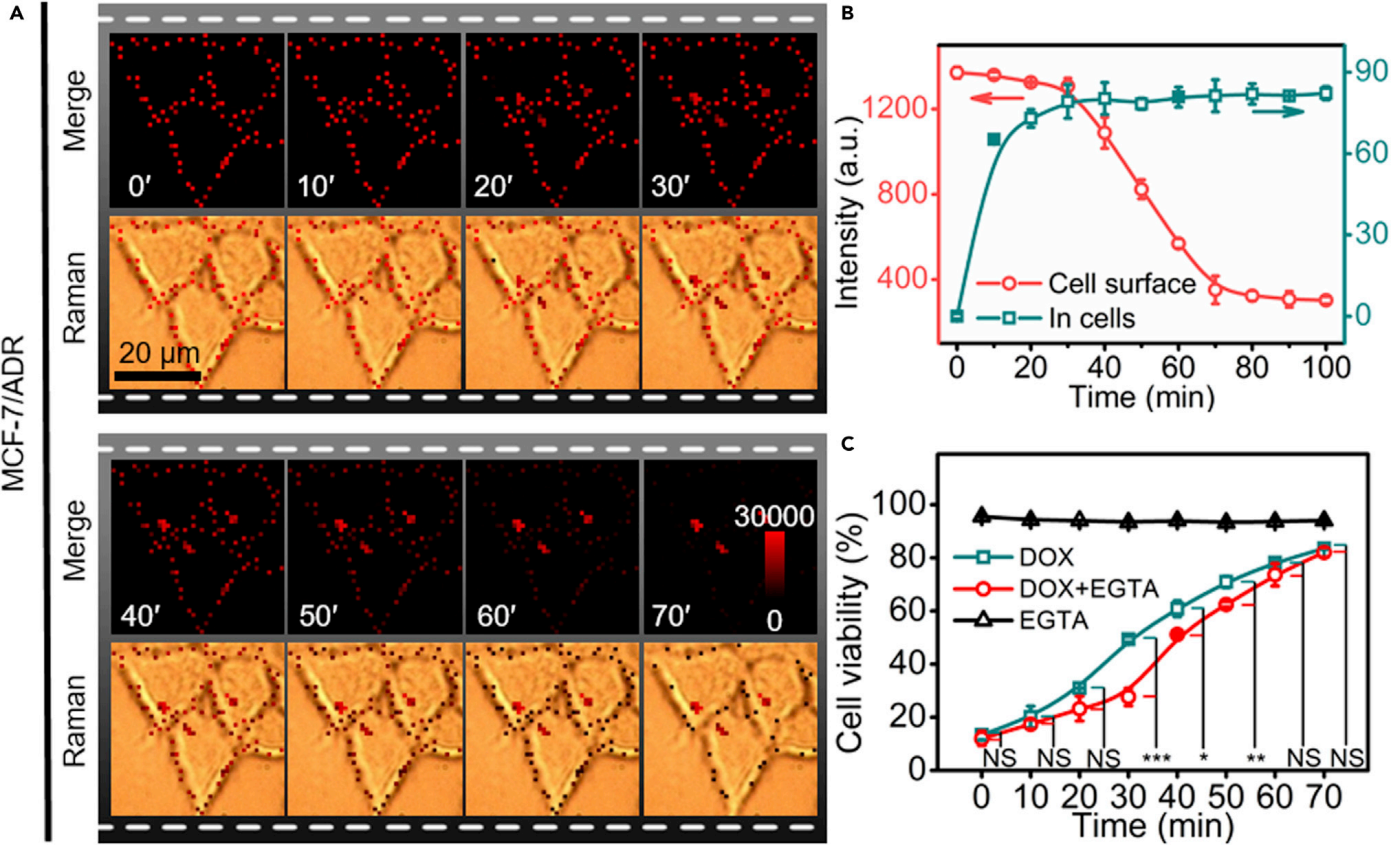
The repair process of perforated MCF-7/ADR cells and blockage of repair for improving drug treatment (A) Dynamic Raman imaging of SLO-DBCO perforated MCF-7/ADR cells after bound with AuNSs-MBA/PEG-N_3_ upon incutaion in FBS-containing RPMI-1640 for 0–70 min. (B) Corresponding statistic signal intensity on cell surface and in cells. (C) CCK8 assay for SLO perforated MCF-7/ADR cells after incubation with 10% FBS-containing RPMI-1640 upon addition of 5 mM EGTA and 1 mM Mg^2+^ (black line), 3 μg mL^−1^ DOX (cyan line) or 3 μg mL^−1^ DOX, 5 mM EGTA and 1 mM Mg^2+^ (red line) every 10 min. The data indicate mean ± s.d. of three independent experiments. Statistical analysis was performed with unpaired two-tailed t test (∗p < 0.05; ∗∗p < 0.01; ∗∗∗p < 0.001; NS, not significant).

Perforation of drug-resistant cancer cells can initiate the drug uptake for disease treatment. The repair of the perforated cell membrane inhibits the efficiency of drug-uptake. Thus, timely blocking of the repairing process is important for highly efficient treatment of drug-resistant cancer cells. Here, DOX-resistant MCF-7 (MCF-7/ADR) cells were used as the model to expand the application of the proposed PIS strategy. The incubation of MCF-7/ADR cells with 100 U mL^−1^ SLO for 10 min, the same treatment as described above for perforation of MCF-7 cells with SLO, could not form the pore on cell surface, while the cell membrane showed obvious pores after the cells were incubated with 300 U mL^−1^ SLO for 20 min (Figures S19A and S19B). Thus, the perforation of drug-resistant cancer cells was more difficult. Once the pores were formed, however, the perforated MCF-7/ADR cell membrane in repairable solution showed faster Raman signal change (Figures 6A and S20) than perforated MCF-7 cells, indicating the repair of perforated MCF-7/ADR cells was easier, which led to shorter time for complete repair than MCF-7 cells (Figures 4C and S19C), though the continuous decrease of Raman signal and the endocytosis of AuNSs-MBA occurred at 30 min (Figure 6B), as that for perforated MCF-7 cells (Figure 4C).

The blocking of the repair process should be performed at the key time of 30 min for obtaining the best treatment of drug-resistant cancer cells. To confirm this viewpoint, EGTA was added into the repairable solution to chelate Ca^2+^ for blocking the repairing process, which was demonstrated by incubating SLO-perforated MCF-7/ADR cells in EGTA-containing repairable solution (Figure S19D). Although EGTA did not change the viability of SLO perforated cells, the addition of EGTA in DOX containing repairable solution to incubate for different times showed obviously lower viability than that in the absence of EGTA (Figure 6C), indicating that the repair of perforated cell membrane inhibited the entrance of DOX to cells, and thus reduced its efficacy. It was worth noting that the maximum difference of cell viability occurred at 30 min, which was well agreed with the time when the Raman signal obviously decreased. Therefore, the PIS strategy could be used for designing the precision therapeutic schedule.

## Discussion

Direct visualization of membrane repair process on injured living cell is very difficult due to the limited sensitivity of imaging methods reported previously. The membrane repair process has the great significance in maintaining cellular homeostasis and ensuring cell survival, and can apply in blocking membrane injury and resisting the entry of extracellular species. This work uses SERS technology to develop a highly sensitive imaging method for tracing the pore-repairing process and probing the membrane repair mechanism on living cell surface.

SLO is firstly selected as the model pore-forming protein to bind DBCO for conjugating MBA-loaded AuNSs via the click reaction with PEG-N_3_ coated on AuNSs (Figure 1), which does not affect the perforating ability of SLO (Figure 2). The formed pores can be directly observed from the AFM and SEM images and repaired gradually by incubating the SLO-DBCO-perforated cells in 10% FBS-containing RPMI-1640 (Figure 2). The *in situ* conjugation of SLO-DBCO on the membrane with AuNS-MBA/PEG-N_3_ brings the strong SERS signals around the formed pores under excitation of both 633 and 785 nm (Figure S7) and maintains the repairable performance of these pores (Figure 2). Furthermore, AuNSs are not internalized into cells during the conjugation and are stable on living cells upon incubation in FBS-free RPMI-1640 to perform an unrepairable process for up to 90 min (Figure S13). Thus, the change of SERS signal can be attributed to the pore repairing process when the cells are incubated in 10% FBS-containing RPMI-1640, which results in the departure of the conjugates of AuNSs-MBA/PEG-N_3_ with SLO-DBCO.

Upon the initiation of membrane repair, the SERS signal firstly decreases very slowly and then continuously reduces to reach the minimum value (Figure 4), which indicates the repair process contains the inspiring and moderate periods. The times of these stages depend on the cell type. In different periods, the directions in which the conjugates have gone are different. In the inspiring period, the repairing process involved both endocytosis and exocytosis of the conjugates, though they are relatively weak, while the latter involved only the exocytosis of the conjugates (Figure 5). Moreover, the exocytosed conjugates cannot be internalized into the cells in repairing medium. Thus, strong Raman signal is directly observed outside the cells. Interestingly, the repaired cells possess the vigorous viability and can be perforated by SLO-DBCO again (Figure S14A).

The SERS strategy shows higher sensitivity than CLSM imaging for dynamic membrane repair process. The latter cannot show the endocytosis signal of the conjugates labeled with AF647 (Figures S16 and S17). Thus, the designed Raman imaging method is a powerful technology for revealing the repairing mechanism.

The sensitive Raman imaging technology shows the universality for monitoring the repairing processes of various cells to perform other applications. For example, the times of the inspiring and moderate periods for HepG2, HeLa, and MCF-7/ADR cells can be evaluated (Figures 6A and S18). The perforated MCF-7/ADR cell membrane shows faster repairing process than MCF-7 cells in repairable solution. By timely blocking the repair process according to the evaluation results, highly efficient treatment of drug-resistant cancer cells has been achieved (Figures 6B and 6C).

In conclusion, the pore-forming protein-induced SERS strategy has been developed for dynamic Raman tracing of cell membrane repairing process on living cells. The high sensitivity of Raman imaging provides a powerful technology with obvious advantage over fluorescence imaging for revealing the repairing mechanism. The exact mechanism of plasma membrane repair has been demonstrated to be mediated by the endocytosis of the conjugates of AuNSs-MBA/PEG-N_3_ with SLO-DBCO in the initial stage and the exocytosis of the conjugates during the whole repair process. Moreover, the exocytosed conjugates cannot be internalized into the cells. The developed strategy possesses the versatile applicability for monitoring the repairing process of different cancer cells and can be expanded to promote the highly efficient treatment of drug-resistant cancer cells by Raman screening of the key time for blocking of the repair process, which facilitates the design of the precision therapeutic schedule.

### Limitations of the study

The cell membrane repairing process on living cells and the corresponding repairing mechanism has been explored using the designed pore-forming protein-induced SERS strategy. The spatiotemporal resolution of the repairing process in this study was limited by the imaging resolution and speed of the confocal Raman microscope.

## STAR★ Methods

### Key resources table

**Table:** 

REAGENT or RESOURCE	SOURCE	IDENTIFIER
**Chemicals, peptides, and recombinant proteins**		

Streptolysin O (SLO)	Sigma	Cat#SAE0089
Chloroauric acid (HAuCl_4_•3H2O, 99%)	Sigma	Cat#G4022
Dithiothreitol (DTT)	Sigma	Cat#D9779
Trisodium citrate	Sigma	Cat#S4641
Propidium iodide (PI)	Sigma	Cat#P4170
Polyvinylpyrrolidone (PVP, MW = 10000)	Sigma	Cat#V900009
Calcein AM	Sigma	Cat#17783
4-Mercaptobenzoic acid (MBA, 99%)	Sigma	Cat#7.6329
Dibenzocyclooctyne-sulfo-N-hydroxysuccinimidyl ester (DBCO-sulfo-NHS ester)	Sigma	Cat#762040
Ethylene glycol-bis(2-aminoethylether)-N,N,N′,N'-tetraacetic acid (EGTA)	Sigma	Cat#324626
Glutaraldehyde	Sigma	Cat#1.04239
RPMI-1640 cell culture medium	KeyGen Biotech.	Cat#KGM31800-500
Dulbecco’s modified eagle’s medium (DMEM)	KeyGen Biotech.	Cat#KG061000
Phosphate buffered saline (PBS)	KeyGen Biotech.	Cat#KGB5001CS
Hank's balanced salt solution (HBSS)	KeyGen Biotech.	Cat#KG025176
Alexa fluor™ 647 nhs ester (AF647-NHS)	Thermo Fisher Scientific	Cat#A37573
Azide polyethylene glycol 2000 thiol (HS-PEG-N_3_)	Shanghai Toyongbio	Cat#P003010-2K
Doxorubicin hydrochloride (DOX)	Aladdin Industrial	Cat#D107159-1g
Fetal bovine serum (FBS)	Thermo Fisher Scientific	Cat#10100147

**Experimental models: Cell lines**		

MCF-7 cells	KeyGen Biotech.	KG031
HeLa cells	KeyGen Biotech.	KG042
HepG2 cells	KeyGen Biotech.	KG020
Adriamycin resistant counterpart MCF/ADR cells	KeyGen Biotech.	KG0311

**Software and algorithms**		

Nanoscope Analysis software 1.80	Bruker	N/A
Renishaw wire 3.4	Renishaw	N/A
Leica application suite x	Leica	N/A

**Other**		

Dimension ICON AFM	Bruker	Dimension ICON
Transmission electron microscope	JEOL	JEM-2100
Scanning electron microscope	JEOL	JSM-7800F
Confocal laser scanning microscope (CLSM)	Leica	TCS SP8
Nanodrop	Thermo Fisher Scientific	2000C
Flow cytometer	Beckman-Coulter	Coulter FC-500
90 Plus/BI-MAS equipment	Brook haven	N/A
MALDI TOF/TOF analyzer	AB sciex	4800 plus
Spectral scanning multimode reader	Thermo Fisher Scientific	Varioskan Flash
Confocal Raman microscope	Renishaw	Renishaw inVia

### Resource availability

#### Lead contact

Further information and requests for resources should be directed to and will be fulfilled by the lead contact, Huangxian Ju (hxju@nju.edu.cn).

#### Materials availability

All materials generated in this study are available from the lead contact without restriction.

### Experimental model and subject details

#### Cell lines

MCF-7, HeLa, HepG2 and MCF-7/ADR cells were obtained and authenticated from the KeyGen Biotech. Co. Ltd. (Nanjing, P. R. China).

#### Cell culturing

MCF-7 cells were cultured in RPMI-1640 media supplemented with 10% FBS, streptomycin (0.1 mg mL^−1^), and penicillin (0.1 mg mL^−1^). HeLa and HepG2 cells were cultured in DMEM supplemented with 10% FBS, streptomycin (0.1 mg mL^−1^), and penicillin (0.1 mg mL^−1^). MCF-7/ADR cells were cultured in RPMI-1640 media supplemented with 20% FBS, streptomycin (0.1 mg mL^−1^), and penicillin (0.1 mg mL^−1^).Cells were grown at 37°C in a humidified atmosphere containing 5% CO_2_ and maintained at densities between 5×10^5^ and 2×10^6^ cells mL^−1^.

### Method details

#### Synthesis and characterization of AuNSs

The synthesis of AuNSs followed the previous method (Kumar et al., 2008). Firstly, 15-nm Au seeds were prepared by adding 5 mL trisodium citrate (1% wt) quickly to 100 mL boiling HAuCl_4_ solution (0.5 mM) to react for 20 min. After the solution was cooled to room temperature, 8.6 mL PVP (25.6 g L^−1^, MW = 10,000) was dropwise added in the solution overnight to form PVP-coated Au seeds. Then, the obtained Au seeds were washed by centrifugation under 12,000 rpm for three times and resuspended in 2 mL ethanol. Next, 43 μL dispersion was added to 15 mL DMF solution containing HAuCl_4_ (0.12 mM) and PVP (10 mM) to react for 15 min. The obtained AuNSs were washed by centrifugation (11,000 rpm, 12 min) and dispersed in 1 mL ethanol. The geometry morphology of the AuNSs was examined with TEM prior to use.

#### Synthesis and characterization of AuNSs-MBA/PEG-N_3_

Before encoding, the obtained AuNSs were dispersed in 1 mL water. 10 μL Raman reporter solution (MBA) (10 mM in ethanol) was then added to 500 μL AuNSs colloids with sufficient mixing, and 490 μL freshly prepared HS-PEG-N_3_ (2 mM) solution was afterward added to the Raman-encoded colloids and shaken at room temperature overnight. Then, the solution was washed by centrifugation under 8000 rpm twice to obtain AuNSs-MBA/PEG-N_3_, which were resuspended in 500 μL water.

The MBA coated on AuNSs-MBA/PEG-N_3_ was evaluated by Raman spectra under 785-nm laser excitation with exposure time of 1s. HS-PEG-N_3_ coated on AuNSs-MBA/PEG-N_3_ was evaluated by DLS and MALDI-TOF MS analysis. For MS analysis, the MALDI-MS spectra were acquired using the reflector mode in the range of *m/z* 1900–2500.

#### AFM and SEM analysis of cells

Cells were seeded in 35-mm plastic plates with glass bottoms. After undergoing adherent growth and the subsequent processing steps, cells were washed three times with PBS, imaged under the AFM or SEM after fixation with 4% glutaraldehyde, gradient dehydration, and natural drying.

For AFM analysis, all images were recorded by force-distance curve-based imaging under PeakForce QNM in Air mode. The AFM image was equipped with a piezoelectric scanner with the scan range up to 90 μm. The commercial AFM cantilevers used in this study had nominal spring constants between 0.05 and 2.3 N m^−1^ and resonance frequencies ranging from 25 to 120 kHz. Properly flatten images were chosen for analyzing the images on the Nanoscope Analysis software 1.80 and measuring the specific topographies through height profiles.

#### Cell perforation with SLO

The perforating process of cells with SLO followed the previously reported protocol (Giles et al., 1998). Since SLO is an oxygen-labile exotoxin, it was activated with 5 mM dithiothreitol at 37°C for 2 h and then stored in small aliquots at −20°C. After washing with HBSS (containing 2 mM CaCl_2_) for three times, the cells were incubated with the activated SLO solution at a final concentration of 100 U mL^−1^ at 37°C for 10 min.

#### Modification of SLO and verification of cell perforation

SLO (0.55 μM) and DBCO-sulfo-NHS ester (47 μM) was mixed to react at room temperature for 2 h. After the reaction was complete, the SLO-DBCO conjugates were concentrated to remove the excess DBCO-sulfo-NHS ester by ultrafiltration (14,000 g, 4°C) using 30 kDa MWCO membranes.

AFM was performed to verify the ability of SLO and SLO-DBCO for cell perforation by incubating the cells with SLO (100 U mL^−1^) at 37°C for 10 min or SLO-DBCO (200 U mL^−1^) at 37°C for 20 min. The AFM images were properly flattened once and then quantified through height profiles across every pore to measure the diameter and depth of every pore.

#### Protein hemolysis assays

50 μL suspension of human erythrocytes (2% in PBS) was added to 0.5-mL sterile Eppendorf tubes containing a certain volume of HBSS and pore-forming protein SLO or SLO-DBCO at different concentrations. Erythrocytes were incubated in HBSS without SLO as a negative control, and in water as a positive control. The hemolysis was allowed to proceed at 37°C for 30 min by inversion. Finally, every tube was centrifuged for 1 min at 200 g. The absorbance values of hemoglobin at 541 nm in the supernatants were assessed to verify the hemolysis of SLO and SLO-DBCO, respectively (Limbago et al., 2000).

#### MALDI-TOF MS characterization of coupling between DBCO and HS-PEG-N_3_

DBCO and HS-PEG-N_3_ with equal concentration were incubated for 30 min at 4°C. 1 μL freshly reaction solution, DBCO, and HS-PEG-N_3_ were then spotted on a 384-well insert Opti-TOF stainless steel MALDI plate (AB Sciex, U.S.A.), respectively, and left to dry. The MALDI-MS spectra were acquired using the reflector mode in the range of *m*/*z* 2000–3000.

#### CLSM imaging of time-dependent perforating process of SLO-DBCO

MCF-7 cells were seeded on four-well confocal dishes and cultured overnight, washed three times with HBSS, and then incubated with PI (10 μg mL^−1^), BSA-containing (1%) HBSS, and a certain dose of SLO-DBCO in a CO_2_ incubator at 37°C for 10, 20 and 30 min, respectively. The cells were then gently washed three times with PBS and imaged by CLSM under excitation at 535 nm. The emission signals were collected from 555 nm to 625 nm.

#### Flow cytometric analysis of cells perforated with SLO-DBCO

MCF-7 cells were seeded on 6-well plates and cultured overnight, washed three times with HBSS, and then incubated with the mixtures of PI (10 μg mL^−1^), BSA-containing (1%) HBSS, and SLO-DBCO at 0–250 U mL^−1^ in a CO_2_ incubator at 37°C for 20 min. The cells were then washed three times with PBS, and the media along with the washing solution were saved in eppendorf tubes, respectively. Afterward, trypsin was added to each well of cells to incubate at 37°C for 1.5 min. The mixtures were saved in corresponding eppendorf tubes and centrifuged (1000 rpm, 8 min) to obtain the cells perforated with different concentrations of SLO-DBCO, which were dispersed in 500 μL PBS (10,000 cells mL^−1^) for FCM analysis.

#### Cytotoxicity of AuNSs-MBA/PEG-N_3_

MCF-7 cells or SLO-DBCO-perforated MCF-7 cells were seeded on 96-well confocal plates and cultured overnight. After three times washing with HBSS, the cells were incubated with different concentrations of AuNSs-MBA/PEG-N_3_ (0–0.6 nM) for 30 min. The cell viability was detected through CCK8 assays according to the instruction.

#### AuNSs-MBA/PEG-N_3_ concentration optimization for reaction with SLO-DBCO-perforated MCF-7 cells

MCF-7 cells were seeded on one-well confocal dishes and cultured overnight, washed three times with HBSS. After perforated with 200 U mL^−1^ SLO-DBCO in HBSS at 37°C CO_2_ incubator for 20 min, the cells were incubated with AuNSs-MBA/PEG-N_3_ (0.2–0.4 nM) for 30 min. Raman images of the cells were acquired to optimize the concentration of AuNSs-MBA/PEG-N_3_ for preventing their endocytosis. The Raman imaging of cells were performed by collecting the MBA peak intensity at 1076 cm^−1^ from the points (1 μm × 1 μm) in an area of 40 μm × 40 μm in signal-to-baseline map review mode with 785-nm excitation at a laser power of 5.2 mW/μm^2^ and an exposure time of 1 s. The focal plane of cells in bright field image was adjusted to give a distinct membrane and inner structure prior to collecting Raman signals. All the data were analyzed with WiRE 3.4 and Origin 2015 software, and the Raman intensity on cell surface was the average of all points.

For SEM imaging of SLO-DBCO-perforated MCF-7 cells after reaction with AuNSs-MBA/PEG-N_3_ at optimized concentration, the slides of cells were treated by spray-platinum and observed under 10.0-kV accelerating voltage and 10.0-mm working distance.

#### Cell damage of different Raman lasers

To examine the laser-scanning-induced damage of MCF-7 cells and SLO-DBCO-perforated MCF-7 cells after incubation with AuNSs-MBA/PEG-N_3_, the cells were scanned with 633-nm laser or 785-nm laser for different Raman mapping times (40–400 min), respectively. The cell viability was detected through CCK8 assays and a live cell staining assay using calcein AM. For fluorescence imaging, the cells were incubated with 2 μM calcein AM for 30 min, then gently washed three times with PBS and imaged by CLSM under excitation at 495 nm. The emission signals were collected from 505 nm to 560 nm.

#### Dynamic Raman tracing of cell membrane repair

After the SLO-DBCO-perforated MCF-7 cells were reacted with 0.2 nM AuNSs-MBA/PEG-N_3_ for 30 min, the cells were firstly imaged with SERS. The treated cells were then incubated with fresh FBS-free RPMI-1640 or 10% FBS-containing RPMI-1640 in a CO_2_ incubator at 37°C and in an interval of 10 min two batches of the treated cells were washed carefully to perform the SERS imaging in FBS-free RPMI-1640 for Raman dynamic monitoring of cell membrane repair. The Raman images were collected from the points (1 μm × 1 μm) in an area of 40 μm × 40 μm.

Considering that the signal inside cells was much weaker than that on cell surface, the statistic signal on cell surface was directly readout through the average of all pixels from Raman images using WiRE 3.4 and Origin 2015 software, which brought negligible influence on the qualitative results, while the weak signal inside cells was the sum of the red channel integration of all cells in the Raman image using Photoshop CS6 software.

The inhibition to cell membrane repair was tested by incubating the MCF-7 cells treated with SLO-DBCO and then AuNSs-MBA/PEG-N_3_ in the mixture of fresh 10% FBS-containing RPMI-1640, 5 mM EGTA, and 1 mM Mg^2+^, in which Ca^2+^ was sheltered by EGTA, to collect the Raman images in FBS-free RPMI-1640 in an interval of 10 min after the treated cells were washed carefully with FBS-free RPMI-1640.

Similarly, SLO-DBCO-perforated HeLa, HepG2, and DOX-resistant MCF-7 (MCF-7/ADR) cells were coupled with AuNSs-MBA/PEG-N_3_ (0.2 nM) for 30 min to perform the Raman dynamic imaging of cell membrane repair.

#### Transmission electron microscopic (TEM) imaging of cells

After the SLO-DBCO perforated MCF-7 cells were reacted with 0.2 nM AuNSs-MBA/PEG-N_3_ for 30 min, the cells were incubated with 10% FBS-containing RPMI-1640 in a CO_2_ incubator at 37°C for 90 min and changed fresh medium every 10 min. The cells were trypsizined and washed with PBS through centrifugation. The formed cell pellet was fixed with 2.5% glutaraldehyde at 4°C overnight. The pellet was then washed with PBS and dehydrated using an ethanol series, embedded in Epon, and sliced with a thickness of 70 nm. Images of the slices were recorded with JEM-2100 transmission electron microscope.

#### Dynamic CLSM imaging for cell membrane repair of SLO-AF647-perforated cells

AF647-labeled SLO (SLO-AF647) was firstly prepared through an amide reaction of SLO (0.55 μM) with AF647-NHS (10 μM) at room temperature for 2 h, and the excess AF647-NHS was removed by ultrafilteration (14,000 g, 4°C) using 30-kDa MWCO membrane. After MCF-7 cells were seeded on four-well confocal dishes overnight and washed three times with HBSS, the cells were incubated with HBSS containing 200 U mL^−1^ SLO-AF647 in a CO_2_ incubator at 37°C for 20 min. The perforated cells were carefully washed with PBS for 3 times and then imaged using CLSM under 651-nm excitation. Afterward, the cells were incubated with FBS-free RPMI-1640 or 10% FBS-containing RPMI-1640 at 37°C for 90 min. During this process, the CLSM imaging was performed in FBS-free RPMI-1640 or FBS-containing RPMI-1640 in an interval of 10 min for dynamic monitoring of cell membrane repair. The fluorescence intensity of cells was acquisited from 660 nm to 700 nm as the average of pixel readouts with Leica Application Suite X.

#### AFM imaging for cell membrane repair of MCF-7/ADR cells perforated with SLO

After the MCF-7/ADR cells were perforated with SLO (300 U mL^−1^) for 20 min in 37°C, they were repaired in 10% FBS-containing RPMI-1640 for 70 min, or incubated in 10% FBS-containing RPMI-1640 with addition of EGTA and Mg^2+^ for 70 min. AFM imaging was used to observe the cell membranes.

#### Cell viability of SLO-perforated MCF-7/ADR cells upon membrane repair

After SLO-perforated MCF-7/ADR cells were repaired in 10% FBS-containing RPMI-1640 for different times, the mixture of 5 mM EGTA and 1 mM Mg^2+^, or DOX (3 μg mL^−1^) or the mixture of 5 mM EGTA, 1 mM Mg^2+^, and 3 μg mL^−1^ DOX was added in the incubation solution to incubate for 3 h. The cell viability was detected with CCK8 assays according to the instruction.

### Quantification and statistical analysis

Experiments were performed using at least three replicates. Statistical significance between two samples was analyzed using unpaired two-tailed t test.

## Data Availability

The published article includes all data generated or analyzed during this study. This study does not report original code. Any additional information required to reanalyze the data reported in this paper is available from the lead contact upon request.
