# Supplementary material for: A pore-forming protein-induced surface-enhanced Raman spectroscopic strategy for dynamic tracing of cell membrane repair
Source: iScience. 2021 Aug 14;24(9):102980. doi: 10.1016/j.isci.2021.102980 (PMC8403736; doi:10.1016/j.isci.2021.102980)
Supplement: Document S1. Figures S1–S20 [file mmc1.pdf]

iScience, Volume *24*

Supplemental information

A pore-forming protein-induced surface-enhanced Raman spectroscopic strategy
for dynamic tracing of cell membrane repair

Yuanjiao Yang, Yunlong Chen, Jingxing Guo, Huipu Liu, and Huangxian Ju

Supplemental Information

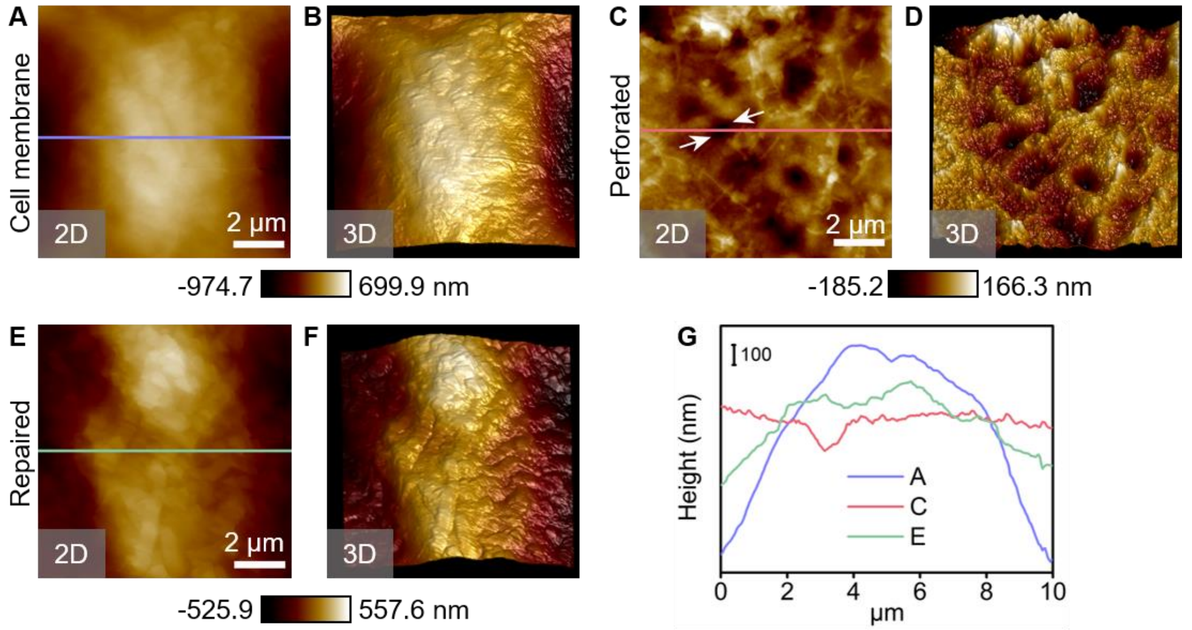

Figure S1. AFM verification of the perforating ability of SLO and repairable ability of cell membrane, Related to Figure 2. AFM images of cell membrane (A,B), cell membrane perforated by 100 U mL^{-1} SLO at 37 ^{o}C for 10 min (C,D) and then repaired in 10% FBS-containing RPMI-1640 for 90 min (E,F). (G) Height profiles corresponding to the lines with the same colors in A, C and E.

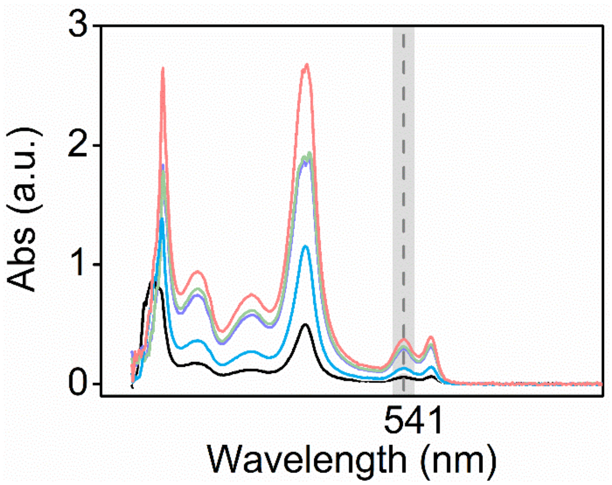

Figure S2. Hemolysis assays verification of perforating ability of SLO-DBCO, Related to Figure 2. UV-vis absorption spectra of human erythrocytes in water as positive control (red), HBSS as negative control (black), and incubated in HBSS containing 100 U mL^{-1} SLO (green) or SLO-DBCO (blue) for 10 min, and 200 U mL^{-1} SLO-DBCO for 20 min (purple).

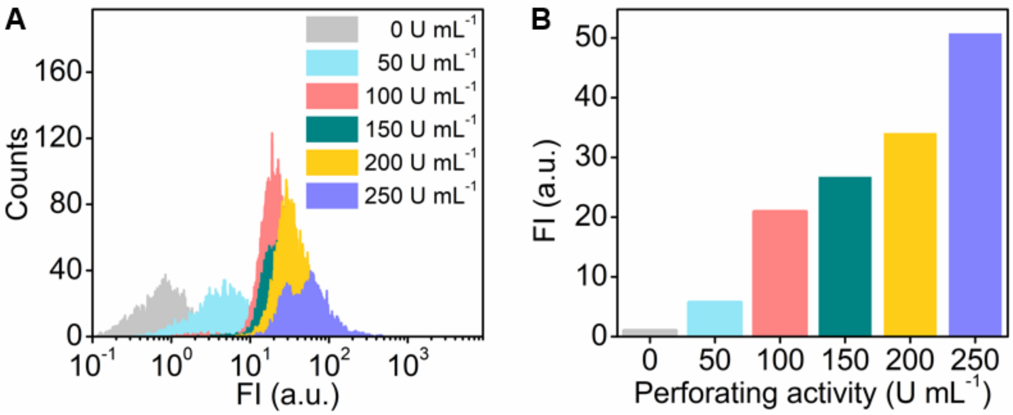

Figure S3. **Optimization of the dose of SLO-DBCO for cell perforation, Related to Figure 2.** (A) Flow cytometric analysis of MCF-7 cells perforated with different doses of SLO-DBCO and then stained with propidium iodide. (B) Corresponding statistic fluorescence intensity. The cells perforated with 250 U mL^{-1} SLO-DBCO showed a cracked peak, indicating excessive cell perforation.

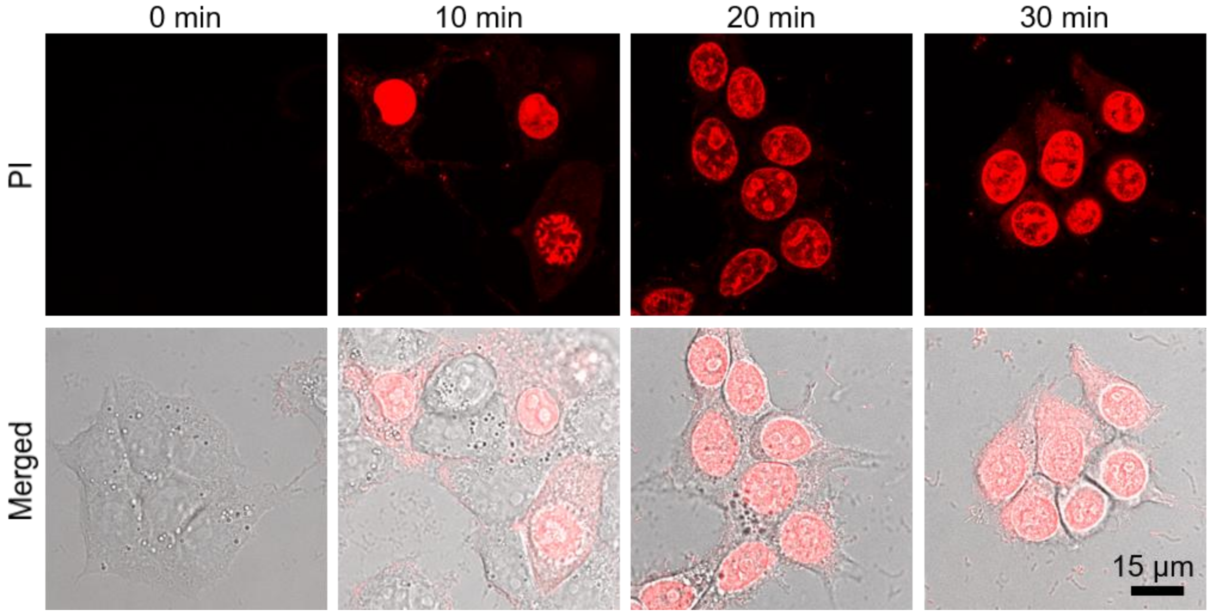

Figure S4. Optimization of the incubation time of SLO-DBCO for cell perforation, Related to Figure 2. CLSM images of MCF-7 cells perforated with SLO-DBCO for different times in the presence of propidium iodide. After the cells were perforated with SLO-DBCO for 30 min, the fluorescence occurred in cytoplasm, indicating excessive cell perforation.

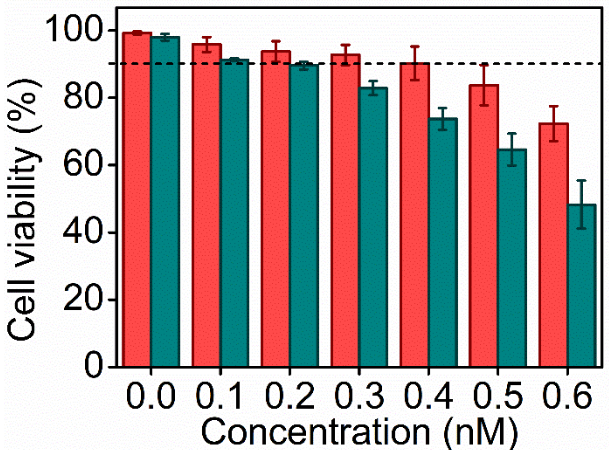

Figure S5. **Cytotoxicity analysis of AuNSs-MBA/PEG-N_3, Related to Figure 3.** Cell viability of MCF-7 cells (red columns) and SLO-DBCO perforated MCF-7 cells (cyan columns) with CCK8 assay after incubation in HBSS with different concentrations of AuNSs-MBA/PEG-N_3 for 30 min. Black dash means cell viability of 90%. The data indicate mean ± s.d. of five independent experiments.

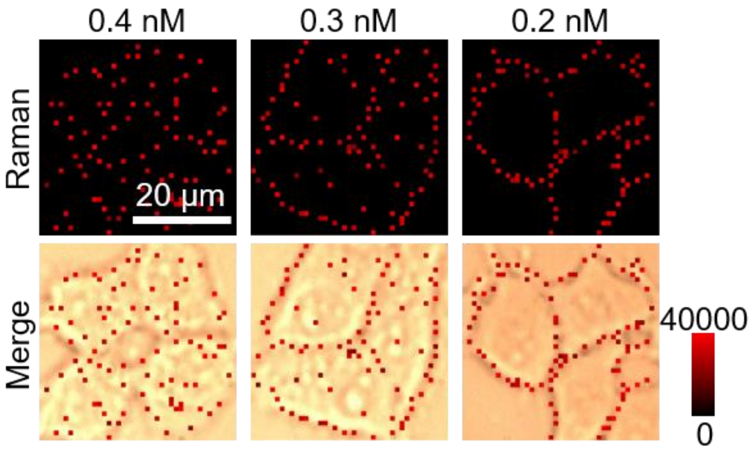

Figure S6. Cell endocytosis analysis of AuNSs-MBA/PEG-N_3, Related to Figure 3. Optimization of AuNSs-MBA/PEG-N_3 concentration for reaction with SLO-DBCO perforated MCF-7 cells to exclude endocytosis. Raman imaging was performed in signal-to-baseline map review mode under 785 nm laser excitation with the peak intensity of MBA at 1076 cm^{-1} from the points (1 μm × 1 μm) in an area of 40 μm × 40 μm.

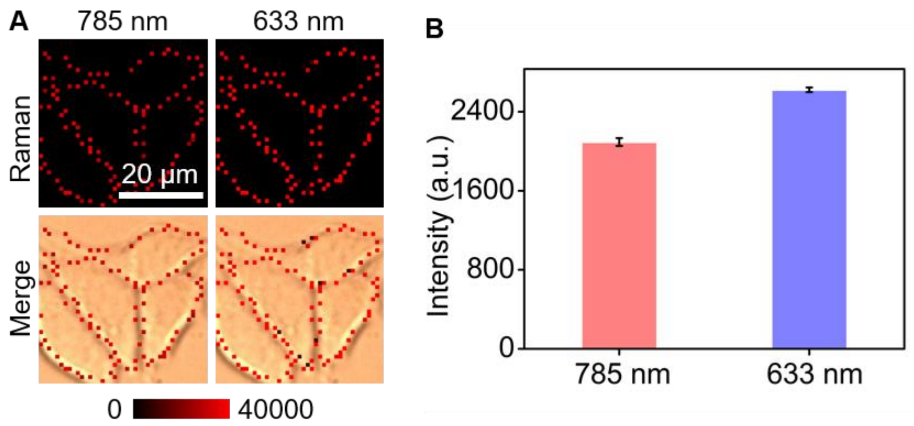

Figure S7. Raman images of MCF-7 cells under different excitation, Related to Figure 4. (A) Raman images of MCF-7 cells under 785 or 633 nm excitation after perforation with SLO-DBCO and then reaction with AuNSs-MBA/PEG-N_3. (B) Corresponding statistic Raman intensity on cell surface. The data indicate mean ± s.d. of three independent experiments.

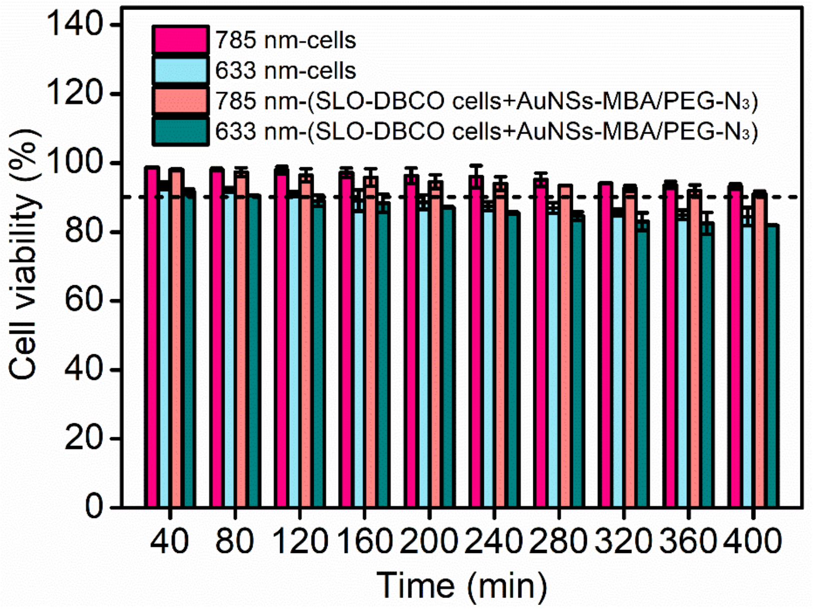

Figure S8. Cell viability after exposure to 785 or 633 nm laser for different Raman mapping times, Related to Figure 4. Cell viability of MCF-7 cells after exposure to 785 nm laser (red columns) or 633 nm laser (orange columns), and SLO-DBCO perforated MCF-7 cells after incubation with AuNSs-MBA/PEG-N_3 and exposure to 785 (blue columns) or 633 nm laser (cyan columns) for different Raman mapping times using CCK8 assay. Black dash means cell viability of 90%. The data indicate mean ± s.d. of five independent experiments.

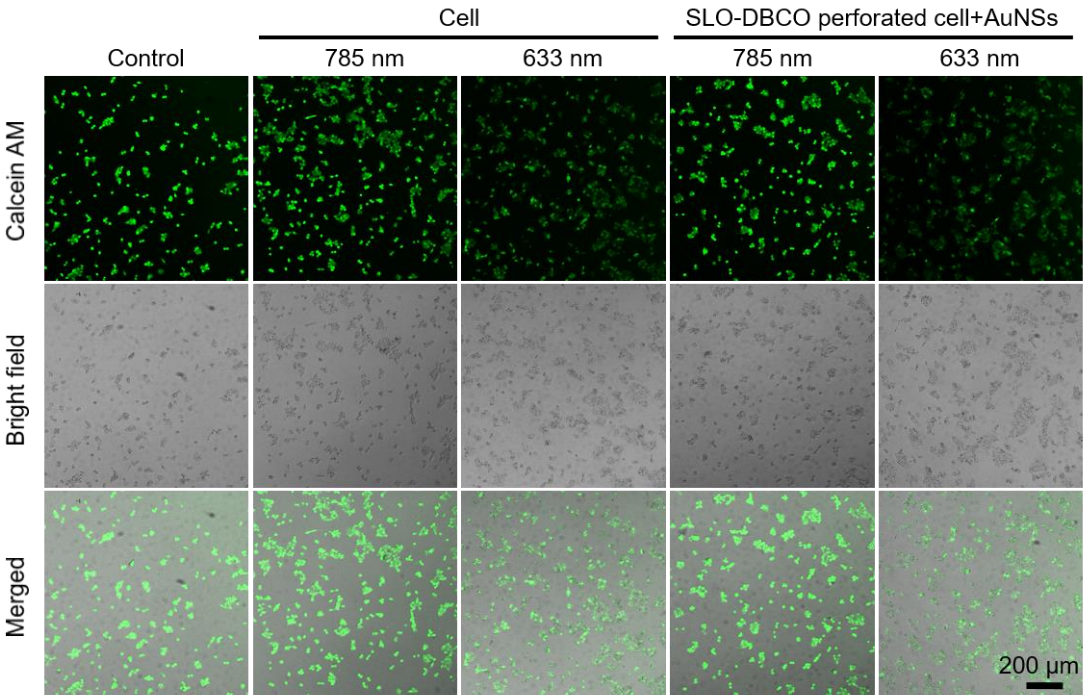

Figure S9. Live cell staining assay analysis of laser damage, Related to Figure 4. CLSM images of MCF-7 cells, and SLO-DBCO perforated MCF-7 cells after incubation with AuNSs-MBA/PEG-N_3 using calcein AM staining, after these cells were respectively exposed to 785 or 633 nm laser for 400 min. Control: untreated MCF-7 cells.

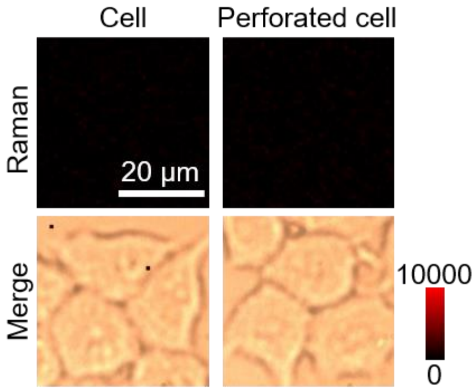

Figure S10. Raman characterization of the aggregation of AuNSs-MBA/PEG-N_3 on cell surface, Related to Figure 4. Raman images of MCF-7 cells and SLO perforated MCF-7 cells after incubation with AuNSs-MBA/PEG-N_3 under 785 nm excitation.

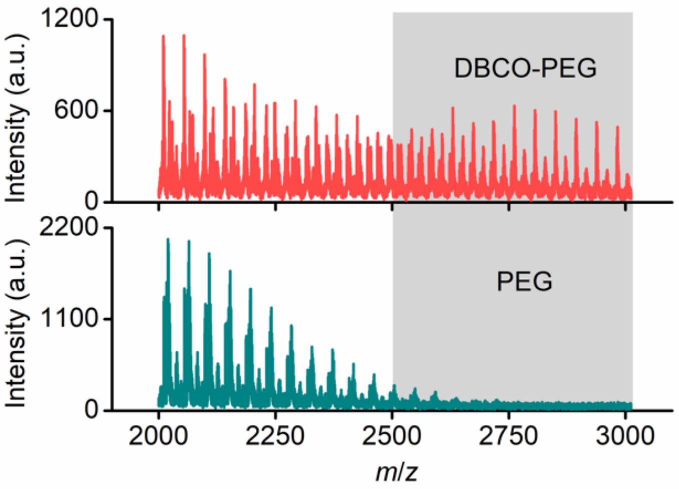

Figure S11. Characterization of the copper-free click reaction between PEG-N_3 and DBCO, Related to Figure 4. MALDI-TOF mass spectra of PEG-N_3-DBCO and PEG-N_3.

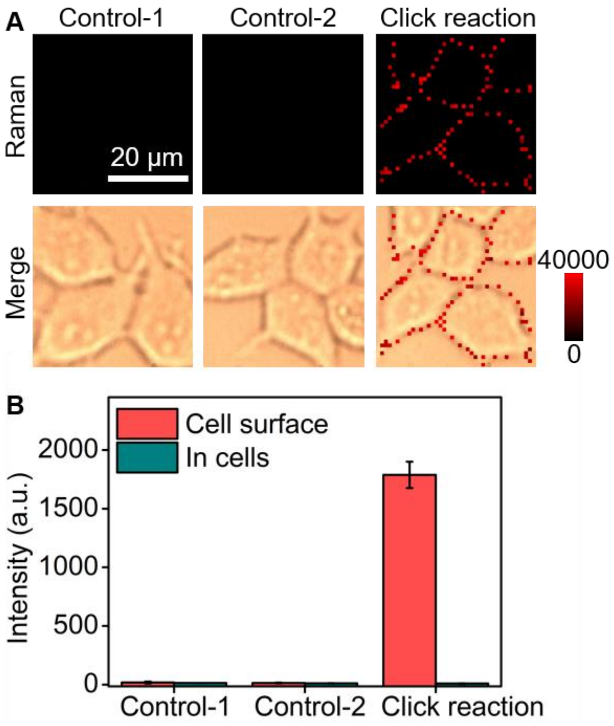

Figure S12. Validation of the copper-free click reaction on cells surface, Related to Figure 4. (A) Raman images of MCF-7 cells after perforation with SLO and then reaction with AuNSs-MBA/PEG-N_3 (Control-1), SLO-DBCO and AuNSs-MBA/PEG (Control-2), and SLO-DBCO and AuNSs-MBA/PEG-N_3 (Click reaction). (B) Corresponding statistic Raman intensity in cells and on cell surface. Raman imaging was performed in signal-to-baseline map review mode under 785 nm excitation using the peak intensity of MBA at 1076 cm^{-1} from the points (1 μm × 1 μm) in an area of 40 μm × 40 μm. The data indicate mean ± s.d. of three independent experiments.

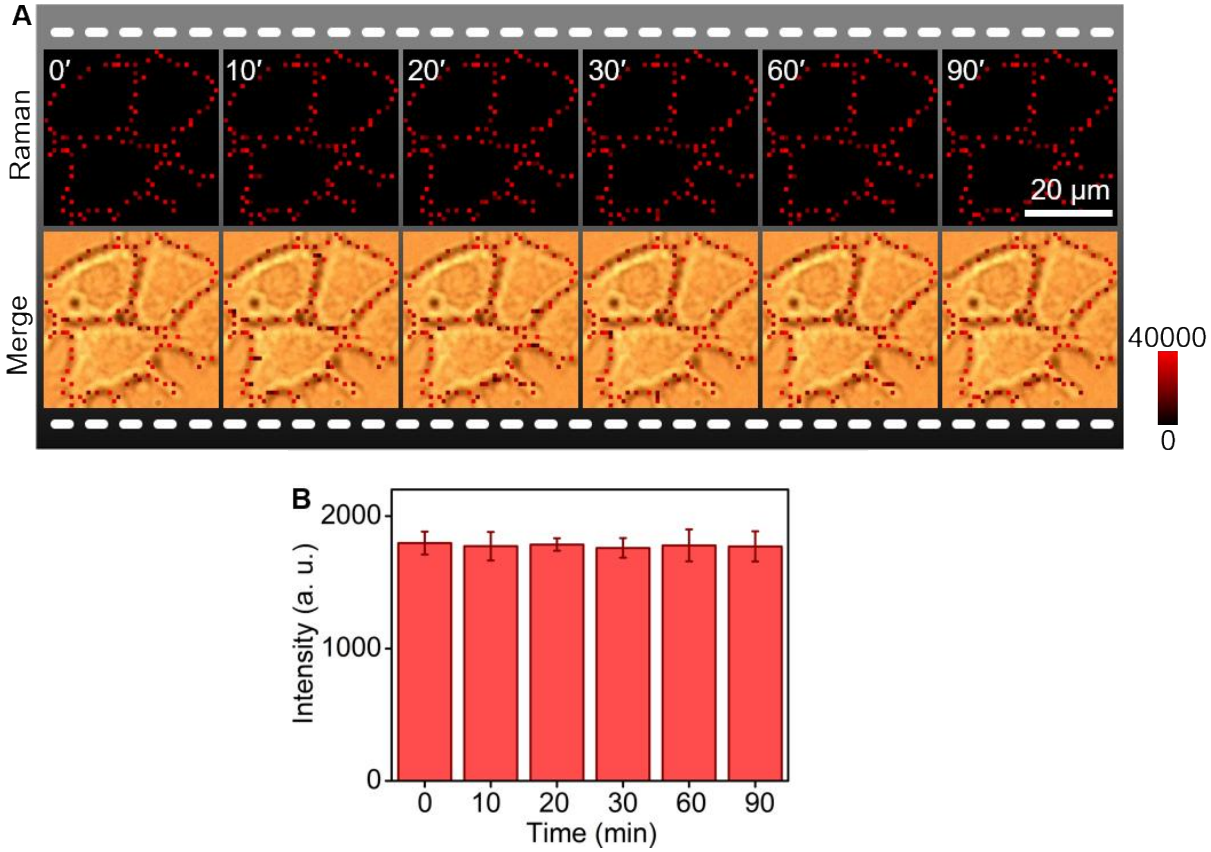

Figure S13. Raman imaging verification of the stability of AuNSs-MBA/PEG-N_3 bound on the cells, Related to Figure 4. (A) Dynamic Raman imaging of the same SLO-DBCO perforated MCF-7 cells after reaction with AuNSs-MBA/PEG-N_3 upon incubation in FBS-free RPMI-1640 for 0 to 90 min. (B) Corresponding statistic Raman intensity. Raman imaging was performed in signal-to-baseline map review mode under 785 nm excitation using the peak intensity of MBA at 1076 cm^{-1} from the points (1 μm × 1 μm) in an area of 40 μm × 40 μm. The data indicate mean ± s.d. of three independent experiments.

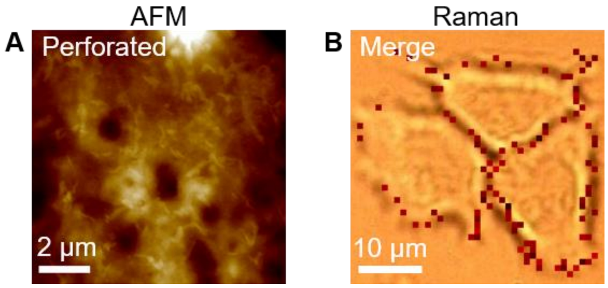

Figure S14. Characterization of the viability of the repaired cells, Related to Figure 4. (A) AFM image and (B) Raman image of the repaired MCF-7 cells after perforation by SLO-DBCO and reaction with AuNSs-MBA/PEG-N_3.

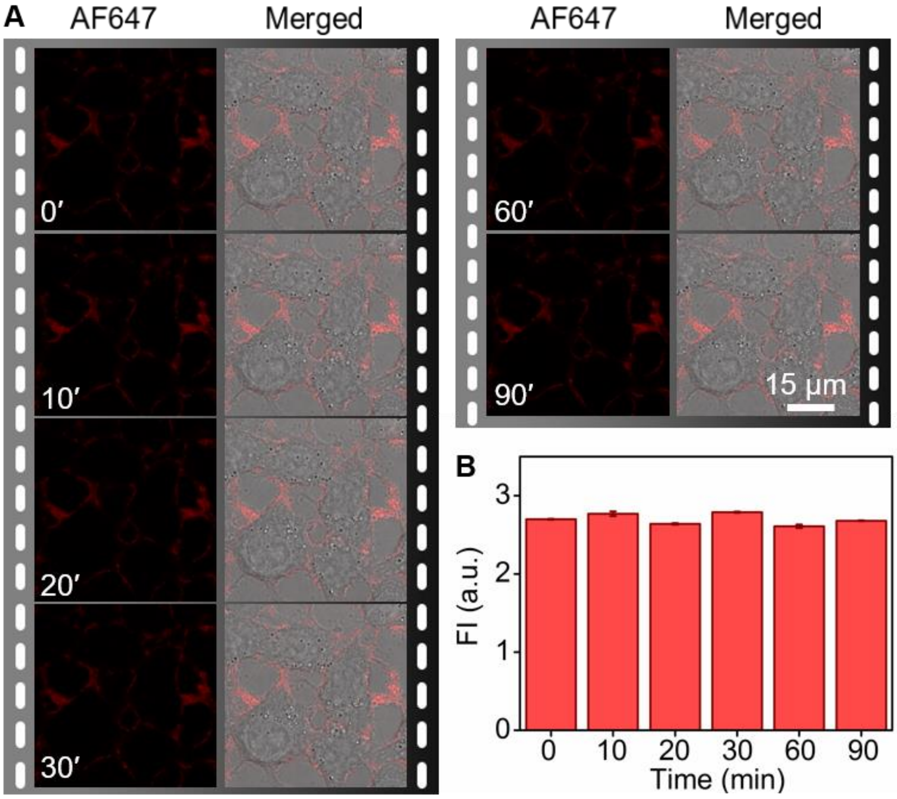

Figure S15. CLSM imaging verification of the stability of signal on cells surface, Related to Figure 4. (A) Dynamic CLSM images of the same SLO-AF647 perforated MCF-7 cells after incubation in FBS-free RPMI-1640 for different times. (B) Corresponding statistic fluorescence intensity. The data indicate mean ± s.d. of three independent experiments.

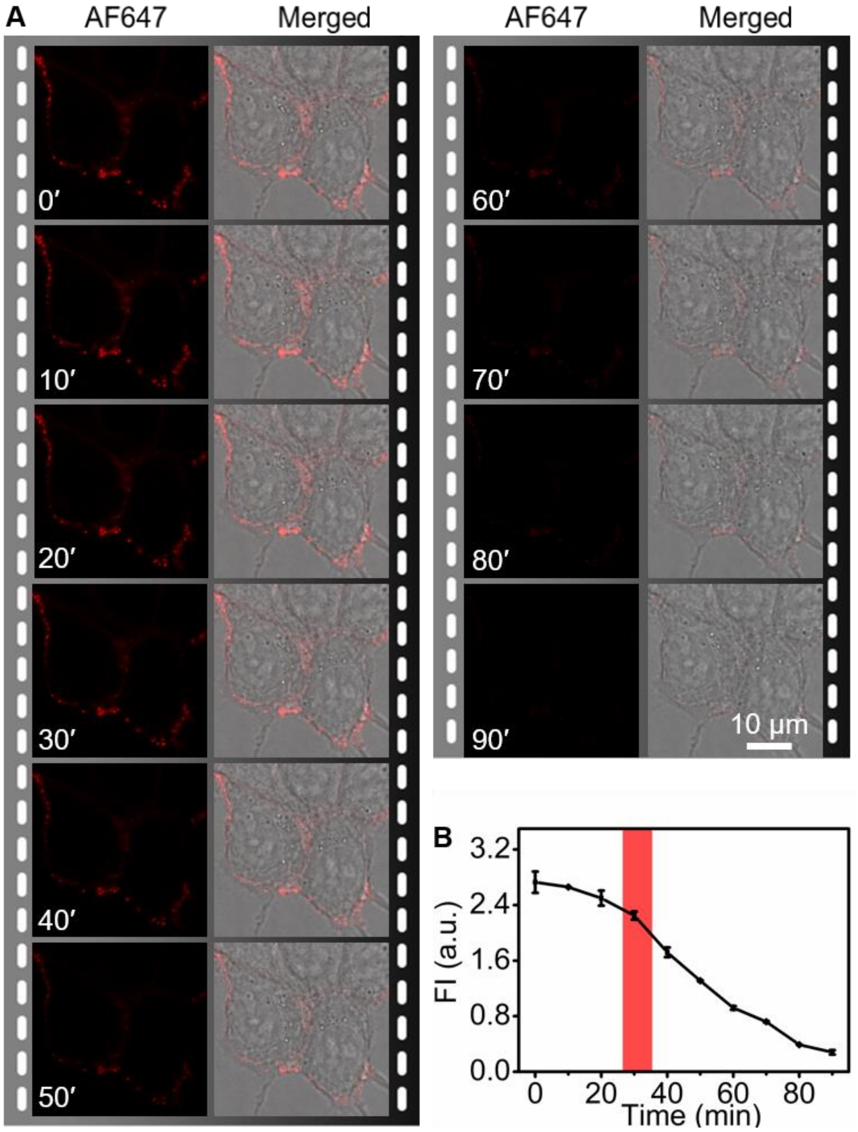

Figure S16. CLSM imaging monitor the dynamic repair process of perforated cells with culture medium exchange, Related to Figure 4. (A) Dynamic CLSM images of the same SLO-AF647 perforated MCF-7 cells after incubation in FBS-containing RPMI-1640 for 0 - 90 min. The imaging is performed in FBS-free RPMI-1640 in every repair interval. (B) Corresponding statistic fluorescence intensity. Red columnar marks FI at 30 min. The data indicate mean ± s.d. of three independent experiments.

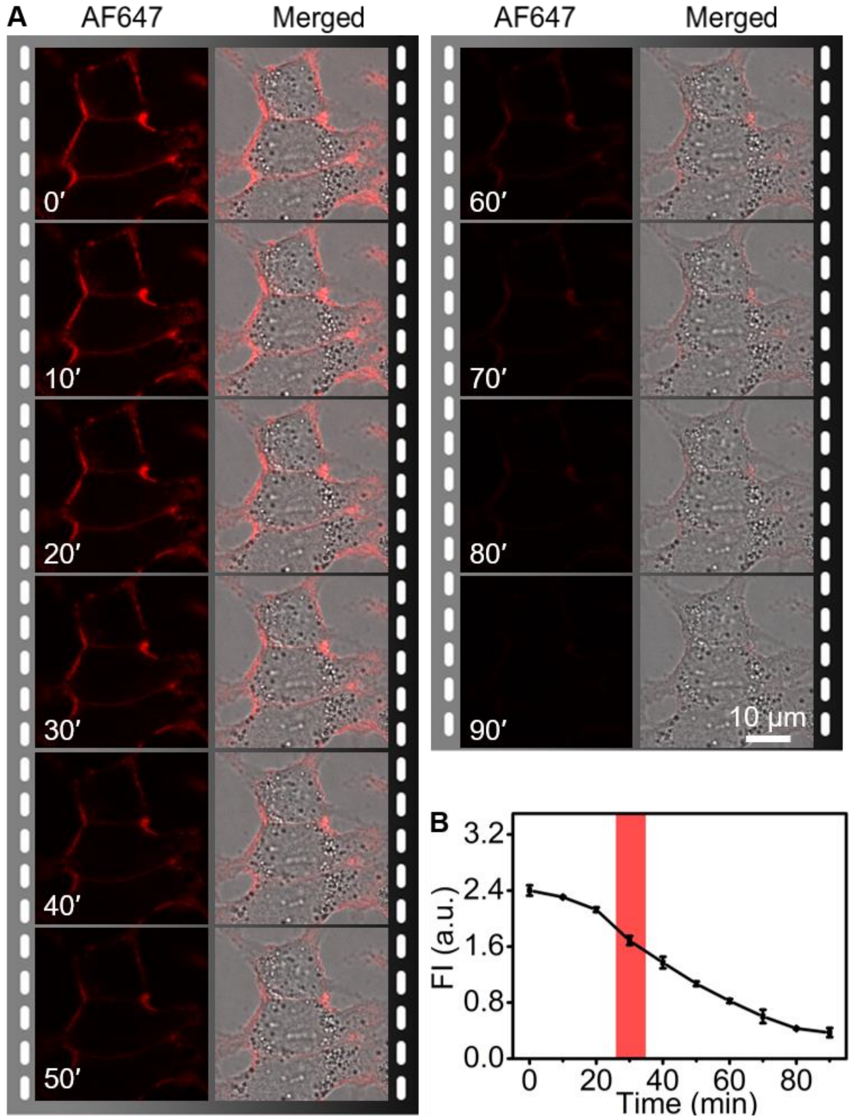

Figure S17. CLSM imaging monitor the dynamic repair process of perforated cells without culture medium exchange, Related to Figure 4. (A) Dynamic CLSM images of the same SLO-AF647 perforated MCF-7 cells after incubation in FBS-containing RPMI-1640 for 0 - 90 min, which are directly taken in FBS-containing RPMI-1640 in every repair interval without culture medium exchange. (B) Corresponding statistic fluorescence intensity. Red columnar marks FI at 30 min. The data indicate mean ± s.d. of three independent experiments.

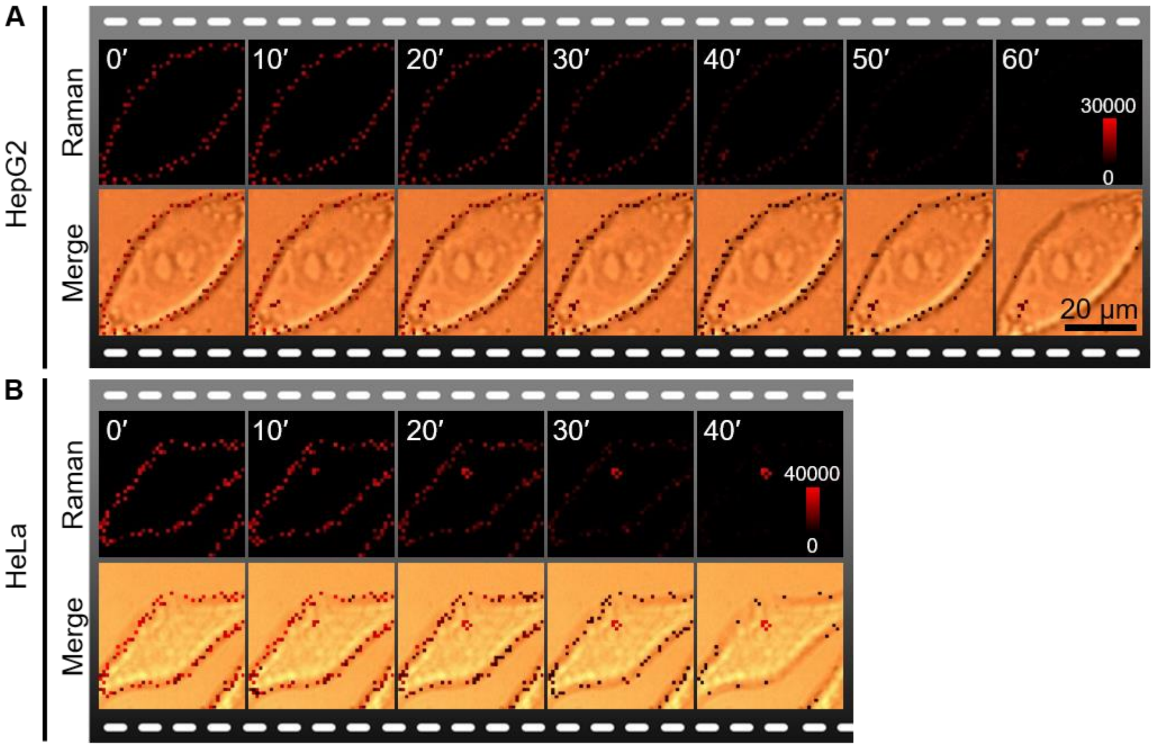

Figure S18. Universality application of Raman imaging strategy for monitoring repair process, Related to Figure 4. Dynamic Raman imaging during cell membrane repair of SLO-DBCO perforated HepG2 (A) and HeLa cells (B) in 10% FBS-containing RPMI-1640 at different times.

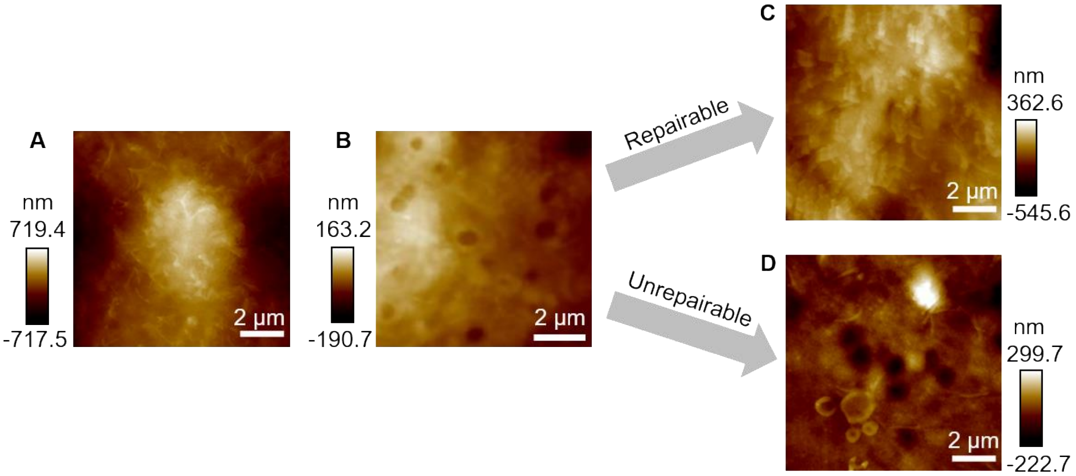

Figure S19. AFM characterization of the perforation and repair of MCF-7/ADR cells, Related to Figure 6. AFM images of MCF-7/ADR cells incubated with (A) 100 U mL^{-1} SLO for 10 min and (B) 300 U mL^{-1} SLO for 20 min. (C) Cell B after incubation in 10% FBS-containing RPMI-1640 for 70 min. (D) Cell B after incubation in 10% FBS-containing RPMI-1640 added with 5 mM EGTA and 1 mM Mg^{2+} for 70 min.

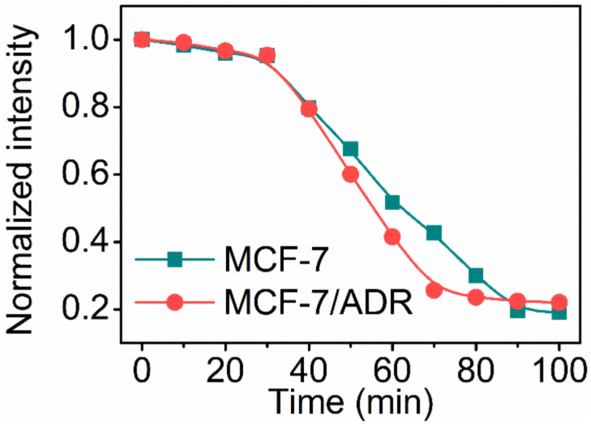

Figure S20. Comparation of the repairable ability of MCF-7 cells and MCF-7/ADR cells, Related to Figure 6. Comparison of dynamic Raman intensity changes in repairing process of SLO-DBCO perforated MCF-7 and MCF-7/ADR cells.
